# Uncovering Genomic Regions Associated With 36 Agro-Morphological Traits in Indian Spring Wheat Using GWAS

**DOI:** 10.3389/fpls.2019.00527

**Published:** 2019-04-25

**Authors:** Sonia Sheoran, Sarika Jaiswal, Deepender Kumar, Nishu Raghav, Ruchika Sharma, Sushma Pawar, Surinder Paul, M. A. Iquebal, Akanksha Jaiswar, Pradeep Sharma, Rajender Singh, C. P. Singh, Arun Gupta, Neeraj Kumar, U. B. Angadi, Anil Rai, G. P. Singh, Dinesh Kumar, Ratan Tiwari

**Affiliations:** ^1^ICAR-Indian Institute of Wheat and Barley Research, Karnal, India; ^2^ICAR-Indian Agricultural Statistics Research Institute, New Delhi, India; ^3^Lokbharti-Sanosara Centre, Bhavnagar, India

**Keywords:** 35K Axiom array, agro-morphological, GWAS, SNP, wheat

## Abstract

Wheat genetic improvement by integration of advanced genomic technologies is one way of improving productivity. To facilitate the breeding of economically important traits in wheat, SNP loci and underlying candidate genes associated with the 36 agro-morphological traits were studied in a diverse panel of 404 genotypes. By using Breeders’ 35K Axiom array in a comprehensive genome-wide association study covering 4364.79 cM of the wheat genome and applying a compressed mixed linear model, a total of 146 SNPs (-log_10_ *P* ≥ 4) were found associated with 23 traits out of 36 traits studied explaining 3.7–47.0% of phenotypic variance. To reveal this a subset of 260 genotypes was characterized phenotypically for six quantitative traits [days to heading (DTH), days to maturity (DTM), plant height (PH), spike length (SL), awn length (Awn_L), and leaf length (Leaf_L)] under five environments. Gene annotations mined ∼38 putative candidate genes which were confirmed using tissue and stage specific gene expression data from RNA Seq. We observed strong co-localized loci for four traits (glume pubescence, SL, PH, and awn color) on chromosome 1B (24.64 cM) annotated five putative candidate genes. This study led to the discovery of hitherto unreported loci for some less explored traits (such as leaf sheath wax, awn attitude, and glume pubescence) besides the refined chromosomal regions of known loci associated with the traits. This study provides valuable information of the genetic loci and their potential genes underlying the traits such as awn characters which are being considered as important contributors toward yield enhancement.

## Introduction

Wheat (*Triticum aestivum* L.) crop provides one-fifth of total food calories and a quarter of protein in the human diet on daily basis ^1^. To meet the increasing food demand of growing population, the breeders focused on the varieties having higher yield and yield stability, increased resistance/tolerance to biotic and abiotic stresses. Approximately 10,000 wheat varieties worldwide ^2^ including 448 wheat varieties in India (Gupta et al., 2018) have been notified. Agro-morphological characterization of germplasm is fundamental in order to provide information for plant breeding programs. The QTL mapping methods based on bi-parental mapping populations identify the genomic regions with low resolution, whereas, Genome-wide association studies (GWAS), based on linkage disequilibrium (LD), take diverse genetic background into consideration to dissect the genetic architecture of complex traits with high resolution. The GWAS in wheat has started gaining importance in the recent past mainly focusing on yield and yield related traits (Liu et al., 2014; Liu Y. et al., 2017; Sukumaran et al., 2014, 2018; Arruda et al., 2015; Gao et al., 2015; Maccaferri et al., 2015; Arora et al., 2017).

Advances in next generation sequencing technology provided valuable wheat genomic and plant breeding resources including high quality genome data (Brenchley et al., 2012; Jia et al., 2013; International Wheat Genome Sequencing Consortium (IWGSC), 2014; Chapman et al., 2015). Several high throughput SNP arrays *viz*., 9K (Cavanagh et al., 2013), 90K (Wang, 2014), 820K (Winfield et al., 2016), 660K (Cui et al., 2017), 35K (Allen et al., 2017), and TaBW280K (Rimbert et al., 2018) have been developed and utilized in wheat. These SNP arrays have been successfully used for GWAS in European winter and spring wheat (Zanke et al., 2014), CIMMYT spring wheat (Sukumaran et al., 2014), United States elite wheat breeding genotypes (Lin et al., 2016), a panel of CIMCOG (CIMMYT Mexico core germplasm) Kazakhstan, Russian, and European wheat genotypes (Turuspekov et al., 2017) and Chinese bread wheat cultivars (Sun et al., 2017). A substantial number of novel SNP variants have been identified using 35K SNP Breeders arrays in the Watkins collection of landraces for further improvement of modern elite cultivars (Winfield et al., 2018). From the 820K SNP array (using global selection of germplasm including elite cultivars, landraces, progenitor, and ancestral species of wheat), Breeder’s 35K Axioms array was developed which contains only mapped SNPs that are tailored to be most informative for specific purposes (Wilkinson et al., 2012; Borrill et al., 2015). 35K SNP array holds promise for detecting large scale variation in secondary and tertiary gene pools (Rasheed et al., 2017).

There are several agro-morphological traits which have been studied intensively and for which markers have been identified (Sukumaran et al., 2018). At the same time there are certain less explored traits for instance awn characters which can be considered as an alternative target for the improvement of wheat grain yield through their known functions including photosynthesis and increased water use efficiency (Rebetzke et al., 2016). The present study includes these characters of future importance hitherto not explored much until now. Moreover, most of the agro-morphological traits undertaken in this study are also utilized for the characterization of the genotypes using Distinctiveness Uniformity and Stability (DUS)^3^.

Some of the studied traits and the associated markers will be of immense importance in future toward developing input use efficient wheat varieties. Amongst them, genomic regions associated with days to heading (DTH) shall enable development of early maturing wheat genotypes to avoid terminal heat stress and allowing an intervening legume crop before rice in the ensuing season (Tewolde et al., 2006; Joshi et al., 2007). For GWAS, size and diversity of the panel plays a significant role as it is suggested that the smaller panel (<384 accessions) and large LD blocks identified in association studies may lead to the identification of false positive associations (Turuspekov et al., 2017). Keeping this in account, a panel of 404 diverse genotypes comprising of indigenous collections, local landraces, released varieties and other improved genotypes was used. The panel also included registered genetic stocks characterized for early maturity, resistance/tolerance to biotic/abiotic stresses, adaptation to different environments, plant architecture etc. (Kundu et al., 2010). These are the major drivers for trait improvement program, using molecular breeding approach. Moreover, India has unique climatic variations rendering wheat germplasm diversity as gold mines. Genotypes adapted to different agro-climatic zones of the country are present in the genotypic panel. Therefore these may be considered as representative of the three major spring wheat growing mega-environments *viz*., ME 1, ME 4, and ME 5 described by the International Maize and Wheat Improvement Center (CIMMYT) spanning across all the 5 continents^4^. This will allow the breeders to utilize the information in developing genotypes specific to different adaptation conditions.

The present GWA study is the first attempt to undertake large scale evaluation of 35K Axiom array in a diverse panel of 404 Indian wheat genotypes. The 35K Breeders’ Array was selected for present work due to its proven efficacy on panel of 1807 accessions of hexaploid wheat (804 accessions from Watkins Collection, a collection of wheat landraces made by A. E. Watkins in the 1920s and 1930s and 1003 modern and elite accessions) from 32 countries (Winfield et al., 2018). The aim of the study was to (i) identify significant MTAs for 36 agro-morphological traits for future breeding and (ii) mining putative candidate genes underlying the corresponding traits of interest. Furthermore, tissue and growth stage-specific gene expression data was also examined providing support to the detected candidate genes. For complex quantitative traits, the association panel was phenotyped at two locations for 2 years. The outcome of this study could be used to make effective strategies for the development of new varieties coupled with economic traits.

## Materials and Methods

### Plant Material

A set of 404 bread wheat (*T. aestivum* L.) genotypes comprising of indigenous collections (91), landraces (8), released varieties (134), genetic stocks (43), and improved genotypes (128) was used to constitute a diverse association panel. The diverse lines were selected on the basis of pedigree, to reduce associations of spurious markers as it provides a buffer against skewness in terms of the environmental effects. Recently, we analyzed trait based diversity analysis using Shannon Index with 16 traits out of 36 traits with a collection of 7,000 diverse germplasm lines (unpublished work). Out of these, 450 genotypes comprising of indigenous collections, landraces, released varieties, improved genotypes and genetic stocks for various traits were selected from 7,000 lines which was downsized to 404 genotypes after eliminating admixtures, duplicates, etc. Comparing the trait based diversity of these selected 404 genotypes using Shannon Index endorsed it as good representative of 7,000 germplasm lines, thereby proving the suitability of 404 genotypes for GWA study (Supplementary Table S1). Seeds of 404 genotypes were obtained from the Germplasm Resource Unit, ICAR-IIWBR (Indian Institute of Wheat and Barley Research), Karnal, Haryana, India, which acts as a nodal center for wheat in the country. Detailed information with pedigree for each genotype is given in Supplementary Table S2.

### Field Trials and Phenotyping

The 404 genotypes were evaluated for 30 qualitative characters at the experimental field of ICAR-IIWBR, Karnal during the crop season 2016–2017. A subset of 260 genotypes was phenotyped for six quantitative traits [days to heading (DTH), days to maturity (DTM), spike length (SL), plant height (PH), awn length (Awn_L), and leaf length (Leaf_L)] at three locations *viz*., Experimental field, Karnal (29°42′N, 77°02′E); Seed Farm, Karnal (29.7138° N, 76.9943°E), Haryana, India and Lokbharti-Sanosara Centre, Bhavnagar (21°46′ N 72°11′ E), Gujarat, India during year 2016–2017. Besides these three environments, an additional environment was taken by phenotyping the subset in crop season 2017–2018 at ICAR-IIWBR, Karnal. Experiment was conducted in two replications following alpha lattice design. To minimize the variations, every genotype was planted with a dibbling tool named IIWBR Dibbler (Sharma et al., 2016) having four rows. The plant to plant distance was 10 cm and row to row distance was maintained at 20 cm. This unique sowing method has helped in avoiding confounding effects of extraneous errors and improved the precision in phenotyping leading to moderate to high estimation of heritability (*H*^2^) thereby enhancing the probability of identifying genes of minor effects related to complex traits (Sharma et al., 2016).

Thirty six agro-morphological characters including coleoptiles anthocyanin coloration (C_Col), plant growth habit (PGH), foliage color (Fol_Col), flag leaf anthocyanin coloration of auricle (Aur_Col), flag leaf hairs on auricle (pubescence) (Aur_Pub), flag leaf attitude (Leaf_Att), ear emergence/days to heading (DTH), flag leaf waxiness of sheath (Wax_LS), flag leaf waxiness of blade (Wax_LB), ear waxiness (Wax_Ear), waxiness of peduncle (Wax_Ped), flag leaf length (Leaf_L), flag leaf breath (width) (Leaf_Br), PH, ear shape (Ear_S), ear density (Ear_D), ear (spike) length (SL), awn presence (Awn_P), awns length (Awn_L), awn color (Awn_Col), awn attitude (Awn_Att), outer glume pubescence (Glu_Pub), ear color (Ear_Col), lower glume shoulder width (Sh_Wid), lower glume: shoulder shape (Shl_Sh), beak length (Beak_L), beak shape (Beak_Sh), spike (peduncle) attitude (Ped_Att), grain coloration with phenol (Grn_Ph), grain color (Grn_Col), grain shape (Grn_Sh) grain germ width (Germ_Wid), brush hair length (Brush_L), seed (grain) size (Grn_Size), grain hardness (texture) (Grn_Tex) and DTM were recorded as per guidelines laid out by Protection of Plant Varieties and Farmers’ Right Authority (PPV and FRA, 2011)^5^. Procedure of recording the data for each trait is summarized in Supplementary File S1. Qualitative traits were recorded as binary (presence or absence), ordinal (visual scale of the expression intensity of a characteristic) and nominal (color or shape) (Supplementary Table S1). For association analysis, a total of five environments (E1–E5) were considered namely, E1 – average of two replications at ICAR-IIWBR, Karnal (2016); E2 – average of two replications at Seed Farm, Karnal (2016); E3–average of two locations of Karnal (2016); E4 – average of two replications at ICAR-IIWBR, Karnal (2017) and E5 – average of two replications at Bhavnagar, Gujarat (2016).

### Statistical Analysis

Phenotypic data was analyzed using SAS v.9.3 (SAS Institute 2011^6^). Pearson pairwise correlation was calculated for all the traits. Histograms were created in R (R Development Core Team, 2013) using the hist() function. The PROC CORR procedure was employed to calculate correlations among phenotypes. Variance components for the quantitative traits were analyzed using general linear model to detect the effect of genotypes, environment, replication and genotype × environment interaction. All sources of variation were considered as random effects. The broad sense heritability for the traits was estimated by the formula *H*^2^ = *V*_G_/(*V*_G_ + *G*_E_) where *V*_G_ and *V*_E_ represent estimates of genetic and environmental variance, respectively.

### SNP Genotyping and Filtering

Genomic DNA was extracted from 15 days old seedlings according to the CIMMYT Molecular Genetics Manual (Dreisigacker et al., 2013). A Nanodrop 1000 spectrophotometer was used for quantifying DNA at 260 nm absorbance (Biodrop Touch PC+125). The DNA samples were used for genotyping with 35K Axiom^®^ Wheat Breeder’s Array (Affymetrix UK Ltd., United Kingdom). Quality preprocessing of 35,143 markers obtained from 35K chip was done by using PLINK software (Purcell et al., 2007). Markers with more than 5% missing values, less than 5% minor allele frequency (MAF) and individuals with more than 15% missing SNP calls were removed from the dataset. Markers with no known chromosomal positions, based on high density consensus map generated by using five mapping populations^7^ (Allen et al., 2017), were also removed. Duplicate markers were further removed by R/QTL software (Broman et al., 2003; Arends et al., 2010).

### Genetic Diversity and Population Structure Analysis

The basic statistics such as genetic diversity (GD) and polymorphism information content (PIC) was evaluated by using PowerMarker v3.2.5 (Liu and Muse, 2005). The model-based Bayesian cluster analysis program, STRUCTURE v2.3.4 (Hubisz et al., 2009) was used to infer the population structure. A total 100,000 burn-in periods followed by 100,000 Markov Chain Monte Carlo (MCMC) iterations from *K* = 2 to *K* = 7 clusters were used to identify the optimal cluster (*K*). Three independent runs were generated for each *K*. The results of the analysis were used as input to the Structure Harvester tool (Earl and VonHoldt, 2011) to predict the best *K*-value based on Evanno method (Evanno et al., 2005). Principal component analysis (PCA) and Neighbor-joining (NJ) tree were created to validate population stratification with the software GAPIT (Lipka et al., 2012) and DARwin v6 (Perrier and Jacquemoud-Collet, 2006), respectively.

### Linkage Disequilibrium

For Linkage disequilibrium analysis, *r*^2^ (squared correlation coefficient) values among markers of all pairs of loci were calculated using PLINK 1.9 tool^8^ (Purcell et al., 2007). Default window size cut off of *r*^2^ value was used for this analysis. Finally, LD plotting was done for three sub genomes (A, B, and D genomes), on the basis of centiMorgans (cM) distance, using ggplot2 package of R Bioconductor (Wickham et al., 2009). The percentage of marker pairs below the critical LD (*r*^2^ > 0.02) was also compared in the sub-genomes. Pairwise LD estimates in the region of interest for significantly associated markers were investigated using Haploview 4.2 (Barrett et al., 2005).

### Association Analysis

Association analysis was performed using compressed mixed linear model (CMLM) implemented by Genomic Association and Prediction Integrated Tool (GAPIT) in R (Lipka et al., 2012) which took into account a *K*-PC model (Zhao et al., 2007) where kinship information together with the first three principal components (PC) as covariates were included for GWAS, which further improves statistical power. Kinship matrix was iteratively calculated using the VanRaden method (VanRaden, 2008). The best fit of the model was evaluated on the Q-Q plots generated by the model. A threshold of -log_10_ *P* > 4 (-log_10_*P* ≥ 4 for quantitative traits) was used to state significant marker trait associations. Associations with false discovery rate (FDR) adjusted at 10% was used to determine the *P*-values thresholds.

### Putative Candidate Gene Analysis and Expression Data

To find candidate genes or putative related proteins of SNP flanking-regions, BLASTx search was conducted for significant MTAs against recently released genome sequence IWGSC RefSeq v1.0^9^. Each MTA was searched for IWGSC sequence information in Ensembl plant for *T. aestivum*^10^. The flanking sequence available for the SNP marker with maximum bases (1,000 bases before and after the SNP) was considered for BLASTx analysis. We also looked at the number of high confidence genes adjacent to the significant MTAs using the RefSeq v1.0.Gene Ontology (GO) annotation of the potential candidate genes was carried out using Blast2GO pro tool v.3.1.3 (Conesa and Götz, 2008). The expression profile of all the putative candidate genes associated with the identified SNPs were checked using wheat RNA-seq expression database of polyploid wheat^11^. This database consists of the transcript profile of five tissues (grain, leaf, root, spike, and stem) at 3 different time points (growth stages) each and environmental treatments (Pearce et al., 2015). Expression of the gene was measured in units of FPKM (Fragments Per Kilobase of transcript per Million mapped reads). Expression profile was carried out to further provide supporting evidence to corroborate candidate genes (tissue and stage of expression).

## Results

### Phenotypic Variation and Correlation Analysis

**FIGURE 1:**
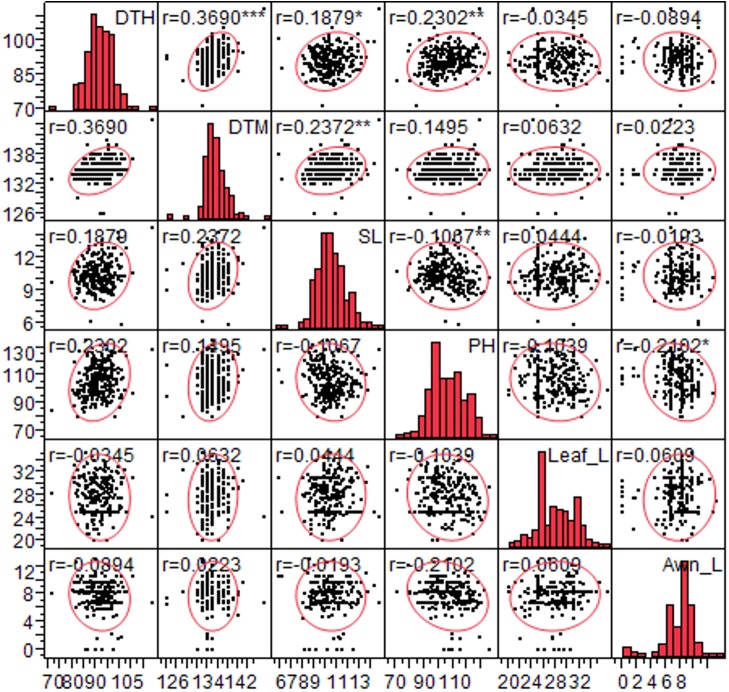
Correlation between six quantitative traits *viz*. Days to heading (DTH), days to maturity (DTM), spike length (SL), plant height (PH), leaf length (Leaf_L) and Awn length (Awn_L) for 260 genotypes. Significant correlations are designated with asterisk (^∗^).

The frequency distribution of phenotypic data of 404 genotypes characterized for 30 traits is given in the Supplementary Figure S1. The phenotypic variations of six quantitative traits (DTH, DTM, PH, SL, Awn_L, and Leaf_L) was recorded in multiple environments. Phenotypic variation of these traits among genotypes was corroborated by mean, standard deviation, range and coefficient of variation (Supplementary Table S3). The mean value of DTH, DTM, PH, SL, Awn_L, Leaf_L varied from 66.72 to 92.0 days, 105.28 to 135.37 days, 94.45 to 113.18 cm, 10.33 to 13.30 cm, 0 to 19.50 cm, 16.25 to 41.40 cm, respectively. This data revealed extensive variation in the traits of the diverse set suggesting the suitability of genotypic panel for association studies. Phenotypic values for each of the six traits were found normally distributed (Supplementary Figure S2). Analysis of variance (ANOVA) was conducted to test the effects of genotype (G), environment (E) and their interactions (G × E). Significant differences were observed among the genotypes (*p* < 0.0001), the effect of environment and their interaction (G × E) indicating the environmental effect on these traits (Supplementary Table S4). Estimates of correlation coefficients of this combined analysis are shown in Supplementary Table S5 and in Figure 1, a positive correlation was observed for DTH with DTM (0.36), SL (0.18), and PH (0.17) while SL exhibited negative correlation with PH (-0.17).

### SNP Markers Statistics

Quality preprocessing of 35,143 markers obtained from 35K chip was done by using PLINK software^12^ (Purcell et al., 2007). 6,041 monomorphic markers were excluded from the analysis. Out of 29,102 SNP markers, 8,673 SNPs failed frequency test (MAF <0.05) and 1,383 markers removed failing missingness test >0.05. Only 2 individuals for low genotyping (MIND >0.2) were removed. Further, 4,740 SNPs were excluded for lack of their physical position and 146 being duplicate markers. After filtering, 402 genotypes with 14,160 SNP markers were used for GWAS. These markers covered a genetic distance of 4364.79 cM, with an average density of 0.3 cM. Marker density was found highest for B genome (1029.6 markers per chromosome) followed by A (788.9 markers per chromosome) and D genome (207.8 markers per chromosome). Among the genome, chromosome 2B had the highest number of markers (1324) while 4D chromosome spanned the lowest number of markers (55) (Supplementary Table S6).

### Population Structure and Linkage Disequilibrium

The mean GD and the PIC for the whole genome were 0.36 and 0.29, respectively. Both GD and PIC of the A genome (0.357 and 0.286) and B genome (0.372 and 0.291) were higher than the D genome (0.345 and 0.276). The number of markers, map length, GD and PIC for each chromosome are shown in Supplementary Table S6.

**FIGURE 2:**
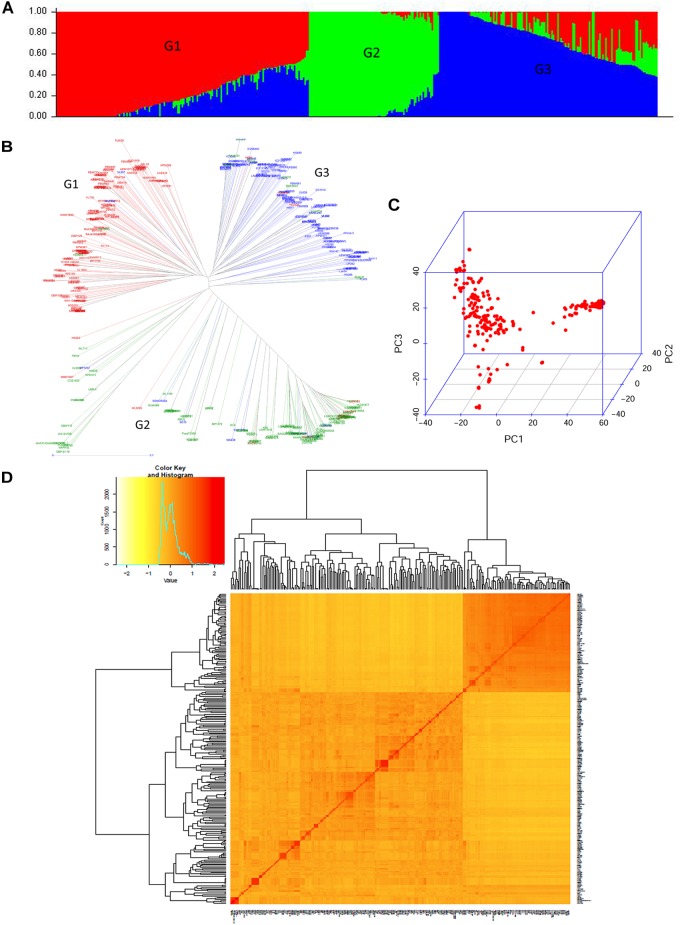
Population structure and diversity analysis of current GWA study panel. **(A)** Population structure based on STRUCTURE when *K* = 3. **(B)** Neighbor-joining based clustering observed in the study panel using 14160 SNP markers. **(C)** Three-dimensional plot of the first three principal components, and **(D)** heat map of pairwise kinship matrix of 402 genotypes.

In the present study, the population structure of a diverse panel of 402 wheat genotypes was investigated on the basis of a Δ*K* method of model-based Bayesian clustering using 14,160 SNP markers. Population structure analysis clearly indicated the existence of three distinct major subpopulations in the bread wheat panel, which was found consistent with the results of the PCA and neighbor-joining (NJ) tree analysis (Figure 2). Subgroup I, the largest group with 169 accessions, was dominated by recently released varieties and breeding lines adapted to Northern wheat growing zone of the country and genetic stocks for biotic resistance (Rust and Karnal bunt). DPW621-50 (2011), HD2967 (2013), WH1105 (2013), HD3059 (2013), DBW88 (2014), HD3086 (2014), and DBW90 (2014) are some recently released varieties. The pedigree showed that the varieties DPW621-50, DBW88, and HD3059 had common pedigree (Supplementary Table S2). Breeding lines for instance HUW675, HUW666, HPW373, HD3133 and varieties MP1201, HS507, HS542 and WH1105 had MILAN in the parentage. Subgroup II consisted of 87 accessions, mainly comprising local landraces from pre green revolution era; Subgroup III had 146 accessions, predominantly from the warmer region of the country and also comprised of early maturing genotypes (short maturity duration of about 120 days) released for late sowing (toward end of November and to mid of December) in different agro climatic zones *viz*., K8962, Raj3765, DBW16, and MP3336 having HD2160 (a triple dwarf genotype) as a common progenitor in their background (Supplementary Table S2). Early Mexican cultivars that paved the way for green revolution, Sonalika, SONORA64, Safed Lerma appeared in this cluster along with the derivatives of Sonalika like UP262, HW2001, and Lok54.

Linkage disequilibrium (LD) decay distance in the selected panel was found highest in the D genome which decayed at about 5 cM (*r*^2^ = 0.02) as compared to ∼2 cM in A and B genomes (Supplementary Figure S3). Faster LD decay in D genome *vis-à-vis* A or B genome has been reported earlier in GWAS of wheat (Lopes et al., 2013; Zhang et al., 2013). With an increase of the genetic distance, the *r*^2^ value of the A, B, and D genomes decreased gradually. Genome A (62.7%) showed the highest frequency of physically linked locus pairs followed by B (58.0%) and D (53.6%) genomes.

### Genome–Wide Association Analysis

**FIGURE 3:**
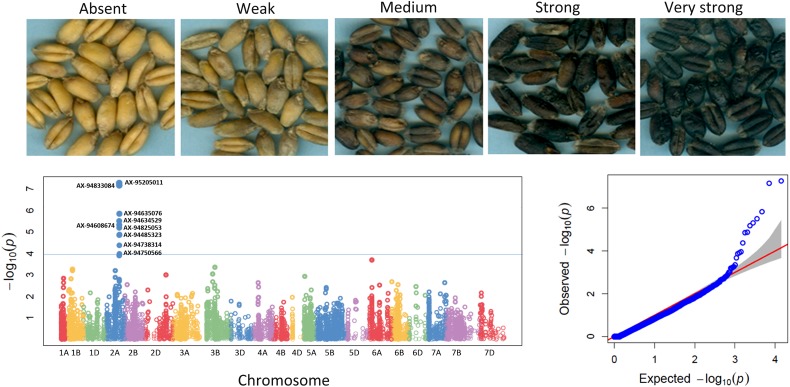
Five different categories of phenol coloration *viz*. absent, weak, medium, strong, and very strong are shown. These variations in coloration are based on the phenol oxidase activity present in the seed coat. Manhattan plot and Q–Q plot for the trait of grain coloration with phenol (Grn_Ph) as observed in the study.

In order to detect the most significant marker-trait associations, CMLM was employed to deal with the confounding effect of the population structure. This was followed by the inspection of Q–Q plots and Manhattan plots for evidence of *P*-value inflation (Supplementary Figures S4, S5). Based on the stringent criterion of -log_10_ *P* > 4, we detected 99 significant MTAs ranging from 7.49 e-05 to 2.47 e-11 for 17 qualitative traits (Figure 3, Table 1, Supplementary Figure S4, and Supplementary Table S7) explaining 5.3–33.3% phenotypic variations. It is imperative to note that, not every gene is likely to be represented by 35K SNP array based markers. Therefore the markers in linkage disequilibrium indicates either it is the causative gene itself or might be in close linkage to the causative gene. For color related traits, a total of 22 SNPs were found associated with coleoptile color on five chromosomes, i.e., 2A, 4B, 6B, 5B, and 6A. However, the genomic region on chromosome 6B was represented by eighteen SNPs, mapped within genetic distance of 62.83–67.99 cM (distance interval of 5.15 cM) which collectively explained 23.9% of the phenotypic variation (Supplementary Figure S4 and Supplementary Table S7). For awn and ear color, a significant MTA was detected on chromosome 1B but at different loci, i.e., at 24.64 cM accounting for a phenotypic variation of 8.8% and at 8.24 cM explaining 10.5% phenotypic variation, respectively (Supplementary Figure S4 and Supplementary Table S7).

**Table 1:** Genome wide significant MTAs identified for the qualitative traits using CMLM.

Traits	SNP	Chr^a^	Position (cM)	Alleles	MAF	*P*-value (CMLM)	Effect	*R*^2^
C_Col^#^	AX-94416850	6B	67.99	T(330)/C(68)	0.1709	2.40E-09	1.064	0.239
	AX-94461579	6B	62.83	A(50)/G(351)	0.1241	1.04E-06	-1.990	0.212
	AX-94673458	5B	52.43	T(349)/C(51)	0.1272	3.23E-06	-0.981	0.207
	AX-95203033	6B	59.6	T(347)/C(51)	0.1278	3.57E-06	-0.946	0.207
	AX-94799212	4B	111.19	T(75)/C(315)	0.1918	5.55E-06	0.609	0.205
	AX-94962809	2A	11.09	A(350)/C(34)	0.0881	8.64E-06	0.928	0.203
	AX-95165934	6A	217.8	C(357)/G(26)	0.0675	9.48E-05	0.862	0.193
PGH	AX-94716155	1B	38.86	C(376)/G(26)	0.0644	5.03E-06	-1.449	0.212
	AX-94873452	1B	38.86	T (374)/C(26)	0.0647	3.12E-05	1.305	0.221
	AX-94940584	1B	8.24	A(322)/G(76)	0.1925	9.47E-05	-1.227	0.20
Aur_Col	AX-94464266	5A	70.36	T(51)/C(346)	0.1278	2.47E-11	1.087	0.223
	AX-94472311	4B	22.53	C(282)/G(114)	0.2889	9.41E-05	1.055	0.152
	AX-94952968	1A	58.79	C(73)/G(328)	0.1811	0.0001	-1.282	0.151
Wax_LS	AX-95000081	6D	177.76	C(100)/G(293)	0.2532	7.49E-05	-0.944	0.164
	AX-94527988	3A	115.9	T(240)/C(160)	0.3980	0.0001	-0.572	0.160
Wax_Ped	AX-94527988	3A	115.9	T(240)/C(160)	0.3980	2.87E-05	-0.665	0.15
Ear_S	AX-94670534	7B	146.84	T(378)/G(21)	0.0524	1.94E-05	-0.099	0.083
Awn_P^#^	AX-94817261	1D	208.95	C(177)/G(221)	0.4425	5.19E-10	-0.294	0.333
	AX-95632082	5B	198.04	A(206)/G(193)	0.4863	2.16E-09	-0.202	0.327
	AX-95109168	7B	1.72	A(241)/G(146)	0.3753	1.03E-08	-0.121	0.321
Awn_Col	AX-95097131	1B	24.64	A(377)/C(24)	0.0596	8.22E-06	0.762	0.088
Awn_Att	AX-94519690	5A	59.99	T(376)/C(22)	0.055	8.51E-06	0.393	0.25
	AX-94613491	5A	70.36	T(374)/C(27)	0.067	8.04E-06	0.357	0.25
	AX-94453668	5A	59.99	T(377)/C(25)	0.0619	6.64E-05	0.338	0.242
Glu_Pub	AX-94506140	1A	50.59	T(49)/C(352)	0.1216	1.47E-07	0.490	0.12
	AX-94626335	1B	24.64	A(370)/G(31)	0.0769	7.77E-06	0.979	0.101
	AX-94618537	1A	75.74	C(309)/G(92)	0.2313	4.62E-05	0.725	0.093
	AX-95255429	2B	76.24	A(353)/G(45)	0.1125	8.36E-05	0.582	0.09
	AX-94852847	2B	76.38	A(355)/G(46)	0.1141	9.40E-05	0.651	0.089
	AX-95023665	2B	104.59	C(348)/G(53)	0.134	4.21E-05	0.743	0.093
Ear_Col	AX-94980178	1B	8.24	A(45)/G(354)	0.1122	5.85E-07	-0.277	0.105
Sh_Wid	AX-94654809	2D	208.85	A(26)/G(363)	0.0665	2.08E-05	1.047	0.147
Grn_ph^#^	AX-95205011	2A	124.89	T(77)/C(324)	0.1911	5.34E-08	-0.785	0.186
	AX-94634529	2A	124.18	T(247)/C(153)	0.3856	3.14E-06	0.676	0.167
	AX-94485323	2A	126.58	T(250)/C(150)	0.3756	1.34E-05	-0.521	0.161
	AX-94738314	2A	124.32	A(139)/T(258)	0.3534	1.39E-05	0.599	0.161
Germ_Wid	AX-94921234	4A	115.15	A(299)/G(100)	0.2494	5.48E-05	-0.598	0.053
Brush_L	AX-94623333	3A	115.9	A(168)/G(231)	0.4214	2.13E-05	0.844	0.206
Grn_Size	AX-95154008	7B	93.52	C (321)/G (79)	0.1965	1.45E-05	-2.448	0.047
	AX-95002032	1A	74.11	A(287)/C(109)	0.2771	9.98e-05	2.545	0.037
Grn_Tex^#^	AX-94471990	6B	62.83	A(347)/C(52)	0.1297	1.54E-05	-1.061	0.085
	AX-95172836	7A	100.09	T(377)/C(23)	0.0572	1.24E-05	-0.591	0.086
	AX-94583333	7A	100.52	A(25)/G(374)	0.0623	6.77E-05	-0.942	0.078


*^*a*^Chr, chromosome, ^#^Only few markers with highest and lowest P-value are mentioned where more SNP markers were associated with trait (C_Col, Awn_P, Grn_Tex, and Grn_Ph). The details of all the markers associated with traits are given in Supplementary Table S7*.

For waxiness characters, two MTAs were identified for leaf sheath wax on chromosomes 6D (7.48 e-05), 3A (*P* < 0.0001) hitherto not reported (Supplementary Figure S4) and one MTA for peduncle wax on chromosome 3A (2.87 e-05). MTAs associated with leaf sheath wax and peduncle wax contributed to the trait negatively. For glume related traits, six MTAs were detected for glume pubescence on chromosomes 1A (2), 1B (1), and 2B (3) explaining phenotypic variation ranging from 8.9 to 12.0% with positive effect. For shoulder width and brush length, significant associations were detected on chromosome 2D and 3A, respectively (Supplementary Figure S4 and Supplementary Table S7). For awn related traits, a genomic region found associated with awn attitude represented by three SNP markers (AX-94613491, AX-94519690, and AX-94453668) on chromosome 5A spanning a region from 59.99 to 70.36 cM. The phenotypic variation explained by SNPs ranged from 24.2 to 25.0% and all the three SNPs showed positive effect on the awn attitude. For awn presence, several markers or regions were identified across the chromosomes (1A, 1B, 1D, 2A, 2B, 3A, 3B, 4A, 4B, 5A, 5B, 6B, 6D, 7A, and 7B) explaining phenotypic variation ranging from 28.7 to 33.9%. A chromosomal region of 12.22 cM (66.99–72.22 cM) on chromosome 5A harbored significant MTAs associated with multiple traits (awn length, auricle color, and awn presence).

For plant growth habit two SNPs were detected on chromosome 1B but at different loci, one at 8.24 cM and other at 38.86 cM indicating the role of two independent loci on chromosome 1B explaining phenotypic variation ranging from 20.0 to 21.2%.

For grain related traits, in the current study 9 SNPs on chromosome 2A spanning 0.71 cM region (124.18–124.89 cM) were found significantly associated with phenol color indicating the importance of this region. These SNPs explained 15.1–18.6% phenotypic variation. For grain texture (phenotype scored as hard or soft), a total of two regions were detected; one on chromosome 7A containing three markers (100.09 cM) and other on chromosome 6B comprising four markers (62.83 cM). Both these MTAs for grain texture contributed negatively to the trait. For germ width, significant association was detected on chromosome 4A. Only one MTA (AX-94670534) for ear shape was detected on chromosome 7B explaining 8.3% of phenotypic variance having negative effect. We did not find any significant MTA for ear density.

For the six quantitative traits (DTH, DTM, PH, Leaf_L, SL, and Awn_L), a total of 47 significant SNPs were identified in five environments which explained 5.2–47.3% of phenotypic variation (Table 2 and Supplementary Figure S5). We successfully detected both previously reported genomic regions and novel loci for the traits in wheat (Tables 1, 2 and Supplementary Figures S4, S5). Flowering time or DTH is a crucial trait which affects the adaptation of wheat in its target environment. A total of 5 SNPs for DTH were detected on chromosome 4A, 5A, and 7D with phenotypic contributions ranging from 19.1 to 32.5%. Out of the 5 SNPs associated with DTH, SNP AX-9454244 on chromosome 4A (78.09 cM) and SNP AX-95187165 (89.02 cM) on chromosome 5A showed pleiotropic effect on DTM. For DTM, a locus on chromosome 2A at 179.61 cM has been detected in the three environments (E2, E3, and E5; Table 2) explaining the average phenotypic variation of 22.9% suggesting the importance of this region while another locus on chromosome 2A at 83.23 cM was observed for three traits (DTM, PH, and SL). Two MTAs were detected on chromosome 5A (AX-95187165, 89.02 cM and AX-95652310, 72.22 cM), indicating the presence of two independent loci on chromosome 5A with a positive effect on DTM.

**Table 2:** Significant MTAs identified for quantitative traits in the current and previous study.

Traits	SNP	Chr^a^	Position (cM)	Physical position (Mb)	Chr (IWGSC)	Allele	MAF	-Log_10_(*P*)	*R*^2^	Effect (Avg.)	Env^b^	Flanking gene locus (Reference)
**DTH**	AX-94542441	4A	78.09	585.03	4A	T/G	0.078	4.29	0.28	-3.52	E5	
	AX-94699167	5A	11.38	586.60	5A	T/C	0.382	4.00	0.28	2.59	E5	9 cM (Sofalian et al., 2008)
	AX-95025549	5A	72.22	–	5B	T/C	0.348	4.00	0.2	-2.87	E5	78 cM (Jaiswal et al., 2016)
	AX-95187165	5A	89.02	412.61	5D	A/G	0.079	4.00	0.2	6.18	E5	90 cM (Sukumaran et al., 2014)
	AX-94508292	7D	127.78	512.75	7D	A/G	0.192	4.00–5.29	0.19–0.32	4.61	E1, E2, E3, E4	176.37 cM (Ain et al., 2015)
**DTM**	AX-95652739	1A	54.04	7.64	1A	A/G	0.283	4.13–4.70	0.07–0.09	-1.81	E2, E3	61 cM (Jaiswal et al., 2016)
	AX-94656878	2A	83.23	88.01	2A	A/C	0.065	4.00	0.09	1.77	E3	76 cM (Jaiswal et al., 2016)
	AX-94463225	2A	179.61	764.09	2A	A/C	0.194	4.65–4.86	0.07–0.10	-2.04	E2, E3	
	AX-94657523	2A	179.61	764.09	2A	A/T	0.162	4.34	0.41	-1.7	E5	
	AX-94681125	3B	61.38	31.74	3A	T/C	0.220	4.04–4.22	0.06–0.09	1.33	E2, E3	62 cM (Jaiswal et al., 2016)
	AX-95115735	3B	84.9	232.56	3B	A/G	0.239	4.00	0.08	-2.23	E3	82 cM (Sukumaran et al., 2014)
	AX-94541758	3B	85.27	219.47	3B	A/G	0.286	5.74–5.75	0.09–0.12	-2.55	E2, E3	82 cM (Sukumaran et al., 2014)
	AX-94542441	4A	78.09	585.03	4A	T/G	0.078	4.15	0.41	-2.25	E5	60.74 cM (Okechukwu, 2017)
	AX-95652310	5A	72.22	–	5A	A/G	0.374	4.00	0.08	1.76	E3	74.2 cM (Gahlaut et al., 2017)
	AX-95187165	5A	89.02	412.61	5D	A/G	0.079	4.00	0.41	3.92	E5	74.2 cM (Gahlaut et al., 2017)
	AX-95229606	6A	78.85	673.01	2A	A/C	0.492	4.09	0.09	1.03	E1	71 cM (Sukumaran et al., 2014)
	AX-95653321	6B	62.83	398.55	6A	A/G	0.471	4.00	0.05	-1.9	E2	50.58 cM (Okechukwu, 2017)
**PH**	AX-95652981	1B	24.64	282.28	1B	A/G	0.49	4.00	0.39	6.07	E4	
	AX-94656878	2A	83.23	88.01	2A	A/C	0.065	4.38	0.38	10.07	E2	Rht7 (Worland, 1996); 88.7 cM (Zanke et al., 2014)
	AX-94841650	2B	104.59	609.30	2D	A/G	0.187	4.00	0.41	-9.42	E5	Rht4, 106 cM (Ellis et al., 2005)
	AX-94737415	5D	1.58	712.00	5B	T/C	0.130	4.05	0.25	4.11	E1	Rht23 (Chen et al., 2015); 0 cM (Jaiswal et al., 2016)
	AX-95170041	5D	1.58	–	5D	T/C	0.137	4.00	0.25	-3.83	E1	Rht23 (Chen et al., 2015); 0 cM (Jaiswal et al., 2016)
	AX-94941145	7A	29.9	19.49	7A	A/G	0.281	4.06	0.47	-6.19	E2	Rht 7, 20.1–29.5 cM (Peng et al., 2011)
**SL**	AX-94981940	1B	24.64	112.20	1B	T/C	0.319	4.21	0.11	-0.96	E5	21 cM (Liu K. et al., 2018)
	AX-94544160	1B	24.64	–	1B	C/G	0.306	4.00	0.1	-0.99	E5	
	AX-94561972	1B	24.64	92.87	1B	A/C	0.266	4.00	0.09	1.77	E5	
	AX-94517196	2A	83.23	528.27	2A	C/G	0.224	4.00	0.16	1.75	E5	
	AX-94702246	2A	83.23	403.21	2D	A/T	0.195	4.03	0.105	1.03	E5	
	AX-94491035	2A	83.23	–	2D	T/G	0.184	4.10	0.104	-1.09	E5	
	AX-95101251	2A	83.23	541.84	2A	T/C	0.197	4.07	0.104	-1.04	E5	
	AX-94539318	3A	84.43	559.54	3A	T/C	0.233	4.00	0.21–0.28	0.92	E3	71 and 73 cM (Sun et al., 2017)
	AX-95252004	3B	85.27	173.62	3D	T/G	0.322	4.22	0.14	-0.84	E2	95 cM (Jaiswal et al., 2016)
	AX-94390305	3D	175.97	813.97	3B	A/T	0.318	4.15	0.14	-0.55	E2	Chu et al., 2008
	AX-94722223	5A	70.36	612.93	7D	T/C	0.308	4.00	0.17	-0.51	E1	
	AX-94976078	7A	24.94	40.87	7D	T/C	0.217	4.49	0.15	0.53	E2	42 cM (Liu K. et al., 2018)
	AX-94551053	7B	1.72	741.57	7B	T/C	0.055	4.00	0.08	-1.06	E5	5.20 cM (Liu K. et al., 2018)
**Awn_L**	AX-94844716	1B	26.22	480.82	1B	T/C	0.065	4.10	0.17	0.75	E1	
	AX-94401263	3B	85.27	581.03	3B	T/G	0.137	4.04	0.17	-0.7	E1	
	AX-94807852	4B	94.44	19.44	4D	A/G	0.090	4.22	0.17	-1.2	E1	
	AX-94895991	4B	96.85	16.92	4D	A/G	0.102	4.08	0.17	-0.75	E1	
	AX-94594057	6D	17.65	507.51	3B	A/C	0.448	4.74	0.18	-0.99	E1	
	AX-94474462	6D	17.65	561.14	6B	A/C	0.414	4.44	0.18	0.86	E1	
	AX-95012310	7B	1.72	634.69	7D	T/C	0.062	6.79	0.20	1.12	E1	
	AX-95025537	7B	2.5	741.57	7B	T/C	0.060	6.86	0.20	1.16	E1	
**Leaf_L**	AX-94675921	4A	214.31	388.34	5D	T/G	0.069	4.58	0.16	-2.01	E4	
	AX-94406861	5A	52.73	692.06	5A	C/G	0.097	4.41	0.16	1.62	E4	
	AX-95196340	7B	24.53	720.37	7B	A/G	0.475	4.10	0.1	1.64	E1	18.91–20.21 cM (Echeverry-Solarte et al., 2015)


*^*a*^Chr, chromosome; ^*b*^Env, environment; DTH, days to heading; DTM, days to maturity; PH, plant height; SL, spike length; Awn_L, Awn length; Leaf_L, leaf length. E1 refers to the average of replications ICAR-IIWBR, Karnal, 2016; E2 refers to Seed Farm, Karnal, E3 refers to the average of two locations of Karnal, 2016; E4 refers to the average of Karnal, 2017; E5 refers to the average of replications Gujarat 2016, respectively*.

For PH, a total of six MTAs were identified, one each on chromosome 1B (24.64 cM), 2A (83.23 cM), 2B (104.59 cM), 7A (29.9 cM), and two on 5D (1.58 cM) considering all the environments (Table 2). MTAs significantly associated with SL were mainly distributed on chromosome 1B, 2A, 3A, 3B, 3D, 5A, 7A, and 7B. The phenotypic variation contributed by SNPs ranging from 8.3 to 27.6% for SL. The SNP AX-94517196 (83.23 cM) on chromosome 2A (Supplementary Figure S5 and Table 2) showed 15.6% of phenotypic variation having positive effect on the trait. Three MTA for flag leaf length were detected on chromosome 4A, 5A, and 7B explaining phenotypic variation ranging from 9.7 to 15.7% (Table 2). The SNP AX-95196340 and AX-94406861 on chromosome 7B and 5A, respectively, showed positive effect on leaf length. For awn length, a total of 8 significant MTAs were identified mainly distributed on chromosome 1B (1), 3B (1), 4B (2), 6D (2), and 7B (2) which collectively explained 18.0% of the phenotypic variation. Two loci, one at 1.72 cM on chromosome 7B and other at 17.65 cM on chromosome 6D shared association with awn length and awn presence.

### Pleiotropy Effect

In the present study, we observed same SNPs with multiple traits which could be due to pleiotropy or different causal genes in LD for instance SNP AX-94656878 at 83.23 cM (chromosome 2A) explained variation for two traits (PH and DTM) (Table 2). Similarly, another locus (SNP AX-94527988) on chromosome 3A at 115.9 cM was found pleiotropic with LS_Wax and Ped_Wax (Table 1). Also, the two SNPs associated with DTH on chromosome 4A (78.09 cM) and 5A (89.02 cM) were found linked with DTM (Table 2). The pleiotropic effects observed in the study were in agreement with the Pearson’s correlations observed between the agronomic traits (Figure 1).

### Identification of Putative Candidate Genes and Expression Analysis

We identified several putative candidate genes such as storage protein activator (spa), beta-amylase 2 (bmy2), cytochrome P450, shikimate kinase, *b-ZIP* transcription factor, for the phenotypic variations of the traits (Table 3). These putative proteins identified were highly homologous to different species of *Triticum* or *Aegilops*. Highest number of putative candidate genes were observed for MTAs associated with SL encoding a total of five candidate genes [actin-related protein subunit 3 (ARPC3), *DIMINUTO*, replication protein A, carboxypeptidase D, and basic region/leucine zipper].

**Table 3:** SNPs significantly associated with agro-morphological traits and putative candidate genes identified in the study.

Traits	SNP	Pos^a^	Chr^b^	Chr _arm IWGSC	Physical position (Mb)	Sequence description/Flanking gene	IWGSC gene ID	Tissue/FPKM^c^	SNP Location
**PGH**	AX-94716155	38.86	1B	1AL	518.81	Storage protein activator	TraesCS1A02G329900	NF	CDS
	AX-94873452			N/A	419.73	MATH domain-containing protein At5g43560-like isoform X2	TraesCS1D02G327700	NF	CDS
**Au_Col**	AX-94464266	70.36	5A	5AL	706.24	Beta-amylase	TraesCS5A02G554200	Grain_z75-1568.43	CDS
**Wax_LS**	AX-94527988^∗^	115.9	3A	3AL	617.07	Cytochrome P450 86A1	TraesCS3A02G368200	<5	CDS
**Wax_Ped**									
**Glu_Pub**	AX-94626335	24.64	1B	1BS	90.99	Metal tolerance protein 1	TraesCS1B02G090400	<5	3′UTR
	AX-94664731			1DL	296.98	Tetratricopeptide repeat protein SKI3	TraesCS1D02G211000	Spike_z39-6.8	CDS
	AX-95023665	76.38	2B	2BL	750.02	Linoleate 9S-lipoxygenase	TraesCS2B02G555400	<5	CDS
	AX-94852847			N/A	72.90	Nicotianamine synthase 9	TraesCS2B02G111100	NF	3′UTR
**Sh_Wid**	AX-94654809	208.85	2D	4AL	80.82	L-type lectin-domain containing receptor kinase IV.1	TraesCS2D02G137300	<5	CDS
**Ear_Col**	AX-94980178	8.24	1B	N/A	4.35	Meiosis_protein_MEI2	TraesCS1B02G008000	NF	CDS
**Awn_Att**	AX-94613491	70.36	5A	4DL	509.67	Hexose carrier protein HEX6	TraesCS4D02G365800	Spike_z39-8.35	CDS
**Brush_L**	AX-94623333	115.9	3A	N/A	478.53	Sialyltransferase-like protein 1	TraesCS3D02G364600	NF	3′UTR
**Grn_Tex**	AX-94471990	62.83	6B	NF	N/A	Metal-nicotianamine transporter YSL2	–	NF	–
	AX-95148325			6DL	320.71	BEACH domain-containing protein C2	TraesCS6D02G229300	<5	CDS
	AX-95086436			NF	500.21	Trehalose-phosphate phosphatase 1	TraesCS6B02G276300	<5	3′UTR
	AX-94583333	100.52	7A	7BL	638.89	ATP-dependent zinc metalloprotease FTSH 2, chloroplastic	TraesCS7B02G373000	Grain_z71-33.49, spike_z65-51.08	3′UTR
**Grn_Ph**	AX-94608674	124.18	2A	NF	568.94	Putative serine/threonine-protein kinase	TraesCS2D02G463100	NF	CDS
	AX-94738314	124.32		2AL	711.08	Probable nucleoredoxin 1-2	TraesCS2A02G466800	Grain_z71-272.53	3′UTR
	AX-94485323	124.58		NF	577.60	APO protein 4, mitochondrial	TraesCS2D02G474900	<5	3′UTR
	AX-94833084	124.89		2BL	690.21	Exopolygalacturonase	TraesCS2B02G491900	<5	CDS
	AX-94635076			NF	574.35	Myosin-3-like	TraesCS2D02G469200	NF	CDS
**DTH**	AX-94699167	11.38	5A	5AL	586.6	Two pore potassium channel a	TraesCS5A02G391400LC	Spike_z32-10.81, stem_z32-9.67	IR
	AX-95025549	72.22	5A	5BS	N/A	OTU domain-containing protein	–	Stem_z30-6.37	–
	AX-94542441^∗^	78.09	4A	4AL	585.03	Shikimate kinase-like protein	TraesCS4A02G276900	Spike_z39-5.21	Intron
**DTM**									
	AX-94656878^∗^	83.23	2A	2AS	88.01	bZIP transcription factor	TraesCS2A02G142800	Spike_z32-33.03	CDS
	AX-94657523	179.61		2AL	764.09	BTB/POZ domain and ankyrin repeat-containing protein NPR1	TraesCS2A02G563400	NF	CDS
	AX-94463225							Spike_z32-13.32	
**PH**	AX-94737415	1.58	5D	5BL	712	Auxin binding protein-1	TraesCS5B02G570100	<5	3′UTR
	AX-94941145	29.9	7A	7AS	19.49	Probable LRR receptor-like serine/threonine-protein kinase At3g47570	TraesCS7A02G042100	<5	CDS
	AX-94656878^∗^	83.23	2A	2AS	88.01	bZIP transcription factor	TraesCS2A02G142800	Spike_z32-33.03	CDS
	AX-94841650	104.59	2B	2DL	609.3	Protein FAR1-RELATED SEQUENCE 5-like	TraesCS2D02G519400	NF	Intron
**SL**	AX-94551053	1.72	7B	7BL	741.57	Cell_elongation_protein_DIMINUTO [delta(24)-sterol reductase]	TraesCS7B02G484200	Spike_z32-42.4	CDS
	AX-94981940	24.64	1B	1BS	112.2	Replication protein A	TraesCS1B02G102200	Spike_z32-6.71	CDS
	AX-94544160				N/A	Carboxypeptidase D	–		
	AX-94561972				92.87	Basic region/leucine zipper protein	TraesCS1B02G091500	Spike_z32-19.42	5′UTR
	AX-94722223	70.36	5A	7DL	612.93	Actin-related protein 2/3 complex subunit 3	TraesCS7D02G509500	Spike_z65-11.84	3′UTR


*^∗^SNP observed in more than one trait; ^*a*^Pos, position; ^*b*^Chr, chromosome; ^*c*^FPKM, fragments per kilobase of transcript per million mapped reads; CDS; coding sequence; IR, intergenic region; UTR, untranslated region; NF, not found*.

To determine the relative expression profile of the identified transcripts in broad range of tissues from different developmental stages, the published RNA-seq data and the Wheat-Exp web tool of the wheat cultivar, Chinese Spring was explored (Choulet et al., 2014; Pearce et al., 2015). The expression profile of significant SNPs encoded putative candidate genes is given in the Table 3 and Supplementary Table S8. FPKM value > 5 was considered for tissue and developmental specific expression check.

### Effect of Favorable Alleles on Agronomic Traits

**FIGURE 4:**
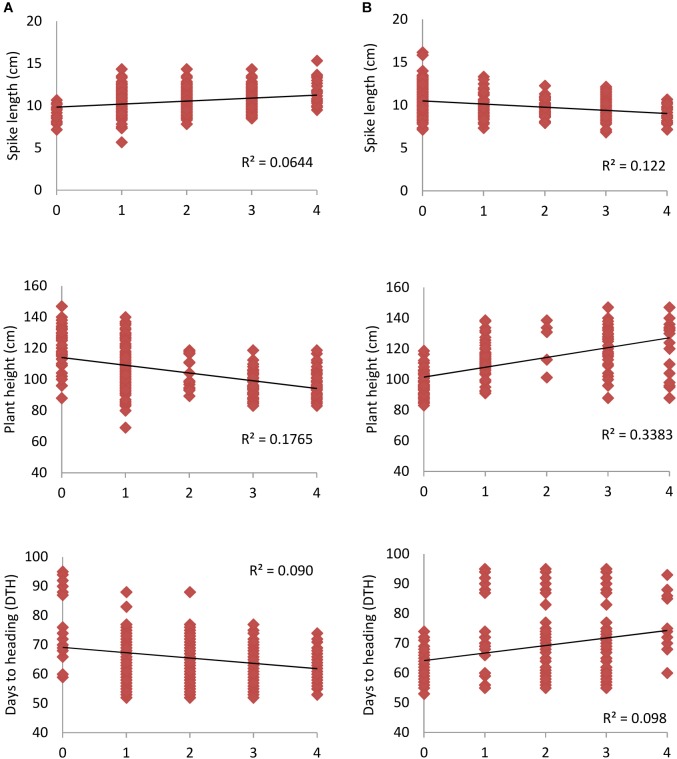
Linear regression between number of **(A)** favorable alleles **(B)** unfavorable alleles for spike length (SL), plant height (PH) and days to heading (DTH). Higher regression value (*R*^2^) was observed in case of PH when compared to SL and DTH for both favorable and unfavorable alleles.

Early maturing, high yielding wheat genotypes are of immense importance toward increasing the cropping intensity as well as ensuring high input use efficiency particularly for inputs like water, which are going to be scarce. Therefore the present study dissecting important agronomic traits such as DTH, PH, and SL enables utilization of available diversity by exploiting associated markers. SNP alleles which led to decrease in DTH, PH and increase in SL were considered as “favorable alleles” and vice-versa was defined as “unfavorable alleles.” Figure 4 depicted higher frequency of favorable alleles which led to decrease in PH and DTH with phenotypic variation of 17.6 and 9.0%, respectively. Similarly, by increasing the number of favorable alleles, SL increased with *R*^2^ of 6.4%. Results of the study showed that favorable alleles exhibited significant positive effects on the phenotypic traits as compared to the unfavorable alleles. This would help in cultivar adaptation and finally to grain yield.

### Traits Sharing Co-localized Genomic Regions

**FIGURE 5:**
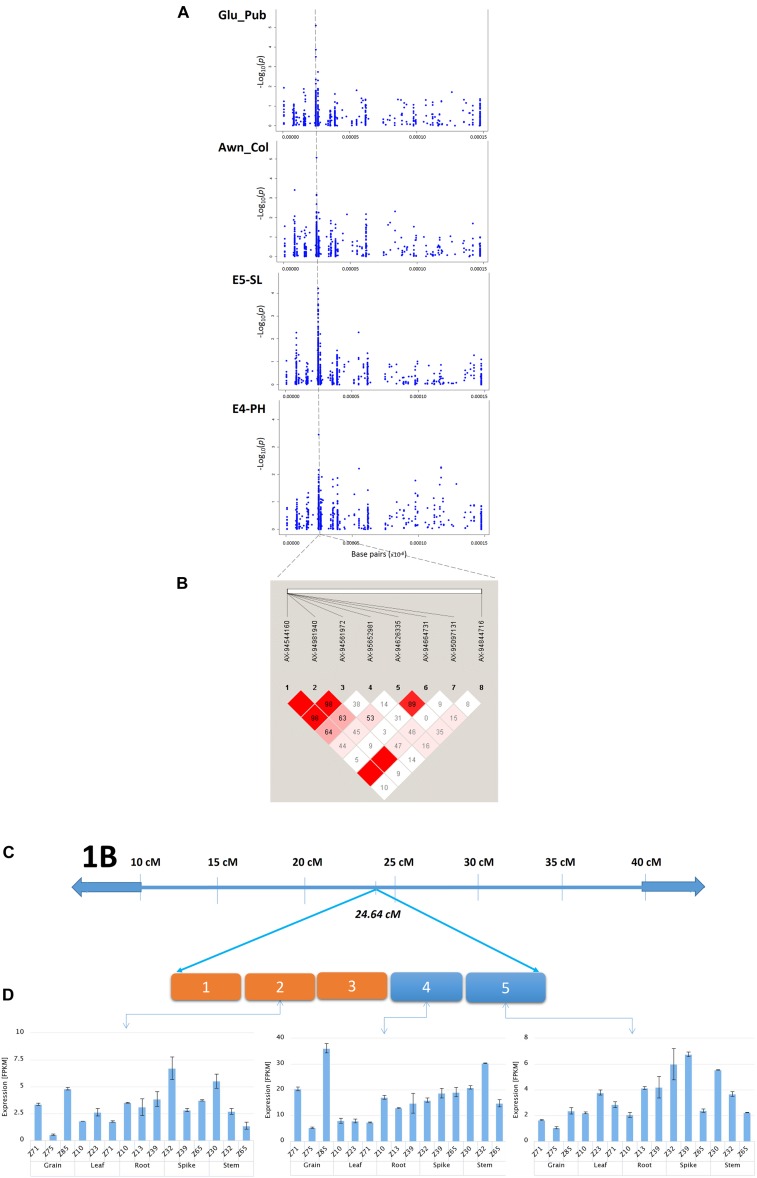
**(A)** Genomic region (24.64 cM) associated with Glu_Pub, Awn_Col, SL, and PH on chromosome 1B as identified by GWAS. **(B)** Pairwise LD estimates in the haplotype block for markers associated with these traits. **(C)** Graphical representation of a chromosome stretch showing putative candidate genes on a locus 24.64 cM (chromosome 1B). Five candidate genes are represented from 1 to 5 [(1) Basic region/leucine zipper protein, (2) replication protein A subunit, (3) carboxypeptidase D, (4) metal tolerance protein, (5) tetratricopeptide repeat protein SKI3]. **(D)** Tissue and stage specific expression profile of candidate genes 2, 4, and 5 (Wheat-Exp database).

In the present study, the most promising co-localized genomic region was identified on chromosome 1B at 24.64 cM associated with four traits (Glu_Pub, Awn_Col, SL, and PH) and 26.22 cM with Awn_L. The genomic co-location of loci (24.64 cM) with four traits implies either a strong physical linkage between genes underlying these important traits, or a pleiotropic effect. Therefore, to dissect the genetic cause of the observed association, LD patterns and candidate genes underlying the region and transcript profile of the targeted region were investigated (Figure 5). The SL and PH at this locus showed greater LD estimates (>0.8) indicating closely dependent biological processes. Whereas comparatively moderate and low LD was observed with Glu_Pub and Awn_Col, respectively, might be due to low overall recombination *vis-à-vis* greater recombination frequency with other genomic regions. Notably, this locus harbored three candidate genes for SL. SNP AX-94981940 (-log_10_ *P* = 4.21), was annotated as a replication protein A subunit and its transcript expression was almost solely abundant in the young spike at Zadoks 32 stage (Table 3). Replication protein A has important role as single strand DNA binding protein in various DNA metabolic pathways (Aklilu and Culligan, 2016). Similarly, the other SNP encoded a protein carboxypeptidase-D which functions as a positive regulator of grain size in rice (Li et al., 2011). The sequence of SNP AX-94561972 linked with SL annotated as basic region/leucine zipper protein. The *bZIP* transcription factor family plays an important role in growth, development, and response to abiotic or biotic stresses (Yin et al., 2017). It is interesting to note that PH shared common significant loci with SL showing high correlation between these traits in concurrence with the previous results (Sukumaran et al., 2014). SNP AX-94626335 (-log_10_ *P* = 5.10), associated for Glu_Pub at this locus (24.64 cM) was annotated as metal tolerance protein (MTP) which is known for its potential involvement in providing a sink for trace element storage in wheat grains (Vatansever et al., 2017). Earlier Echeverry-Solarte et al. (2015) also reported the influence of glume pubescence on SL by identification of a cluster of co-localizing QTL on same locus for both the traits. Another Glu_Pub associated SNP AX-94664731 on chromosome 1B at 24.64 cM annotated tetratricopeptide repeat protein SKI3, showed highest expression at Zadok39 growth stage of spike. The *TaFlo2-A1* gene, an orthologous of rice *Flo2* has four motifs of tetratricopeptides found associated with thousand grain weight (Sajjad et al., 2017) and F-box protein containing domains of tetratricopeptides known to regulate plant development and their abundance during spike development in wheat (Hong et al., 2012). The expression patterns of the putative candidate genes in different organs (Figure 5) are consistent with the RNA-seq FPKM expression patterns. The single genomic locus identified for these important related traits, needs further studies to fine map and validate the identity of the causal locus.

## Discussion

The diversity panel selected in this study has high GD (0.363) and PIC (0.29) indicating higher polymorphism than listed in the previous reports (Liu J. et al., 2017; Eltaher et al., 2018). Further, the B genome had higher GD and PIC followed by A and D genome, consistent with the previous report (Ain et al., 2015). The highest LD decay rate of 5 cM for D genome obtained in this study employing 14,160 SNP markers was found in congruence with 90K SNP (Sukumaran et al., 2014) and 9K (Lopes et al., 2013) marker data. The results from the three clustering methods (Structure, PCA, and NJ tree analysis) showed the presence of three subpopulations in this study consistent with the geographic origins and pedigrees of the selected panel. Thus the marker density, diversity and sample size of this study is sufficiently powered to capture allelic variations for the selected traits. Ma et al. (2013) reported that various imputation methods could be used to impute the data from low density to high density, i.e., from 3K to 54K, and subsequently from 54K to 777K. Therefore the data generated with 35K breeders array can be imputed to high density using 820K information.

In this study, a GWAS panel was characterized for 36 agro-morphological traits identified 146 MTAs (-log_10_ *P* ≥ 4) for 23 traits. For majority of the heritable traits, at high significant level single locus has been identified indicating that they are controlled by small number of loci, for instance PGH (1B), LS_wax (6D, 3A), Ped_Wax (3A), Sh_Wid (2D), Ear_Col (1B), Ear_Sh (7B), Awn_Col (1B), Awn_Att (5A), Brush_L (3A), Germ_Wid (4A), and Grn_Ph (2A). For coleoptile color, the genes that regulate anthocyanin biosynthesis pathway, have been cloned and mapped on homoeologous groups 3 and 6 (phenylalanine ammonia-lyase), homoeologous groups 1 and 2 (chalcone synthase), homoeologous group 5 (chalcone-flavanone isomerase) (Li et al., 1999). The presence of the dominant allele at the *Rc-1* homeologous loci responsible for anthocyanin pigmentation in coleoptile was correlated with *F3H* (*flavanone 3-hydroxylase*) gene on chromosome 2A (Khlestkina et al., 2008). However, in this study, besides chromosome 2A, we also detected the loci for coleoptile color on chromosome 6B, 5B, 4B, and 6A. In agreement to this, Sutka (1977) identified the gene designated *Rc4* for coleoptile color on chromosome 6B, however, it was not further confirmed in any study (Khlestkina et al., 2002; McIntosh et al., 2014) while suppressors playing role in the intensity of the coleoptile coloration were identified on chromosomes 2A, 2B, 2D, 4B, and 6A of “Mironovskaya 808” bread wheat variety. Hence the loci identified herein further confirmed the role of chromosome 6B for coleoptile color. For Awn_Col and Ear_Col, we identified loci on chromosome 1B but at different positions, i.e., 24.64 and 8.24 cM, respectively. Earlier, Zeven (1983) also reported a semi-dominant gene (*Rg*) on chromosome 1B responsible for the brown ear character of bread wheat. For Aur_Col, contrasting to the region reported on chromosome 4A and 5B (Zeven, 1985), we detected its locus on chromosomes 4B and 5A owing to the instability of its expression.

Epicuticular wax is associated with increased drought tolerance in wheat (Bennett and Schnurbusch, 2016), rice (Haque et al., 1992), maize (Meeks et al., 2012), barley (Febrero et al., 1998), and many other crops. Herein, we report an additional locus for LS_Wax on chromosome 6D besides previously identified genomic region on chromosome 3A for waxiness. Interestingly, the SNP AX-94527988 (chromosome 3A) was found associated with both LS_wax and Ped_wax thereby indicating its pleiotropic behavior. In fact, this result indicated that some casual gene(s) might exist in this genomic region for wax, as the common MTA AX-94527988 annotated cytochrome P450 protein which leads to a double-hydroxylation to the corresponding oxo-2-alkanol esters which are also previously detected for both peduncle and flag leaf waxes (Racovita et al., 2016).

Glume pubescence appears to have a beneficial influence on drought/cold tolerance (Borner et al., 2005). In this study, we identified significant MTA (1.47 e-07) on chromosome 1A which is in agreement with Sears (1953) who mapped a gene (*Hg*) responsible for Glu_Pub on chromosomes 1A. In addition to this, we detected genomic regions associated with Glu_Pub on chromosomes 1B (24.64 cM) and 2B (76.24, 76.38, and 104.59 cM) which might be considered as novel region controlling the trait. MTA AX-95023665 linked to Glu_Pub encoded fatty acid biosynthetic process. Glas et al. (2012) observed that methyl ketones which are produced during fatty acid biosynthesis were the major constituent of type VI trichomes of the wild tomato *Solanum habrochaites f. glabratum* and are very effective in protecting the plant from pests. Pubescent plants also produced a higher number of grains per spikelet compared to non-pubescent plants (Maes et al., 2001).

Several MTAs detected for awn presence were found distributed across the wheat chromosomes except for 2D, 3D, 4D, 5D, 6A, and 7D. However, we could not locate any awn development dominant inhibitor genes *Hd, B1, B2* fine mapped on chromosome 4AS, 5A, and 6BL in hexaploid wheat (Sourdille et al., 2002; Yoshioka et al., 2017) which may be due to skewness for the awned genotypes in the diversity panel. For awn attitude, one QTL located on chromosome 5A (59.99–70.36 cM) was identified. To the best of our knowledge, none of the previous studies have reported a genomic region for Awn_Att, suggesting this could be a responsible locus for the trait. The MTA AX-94613491 encoded hexose carrier protein HEX 6 which is responsible for controlling the flux of carbon and plays a role in the carbohydrate transport and distribution in plant cells^13^.

A gene for high PPO activity responsible for grain color was mapped on the long arm of chromosome 2A in wheat mapping population (Simeone et al., 2002). Similarly, in the current study, 9 SNPs on chromosome 2A spanning 0.71 cM region (124.18–124.89 cM) were found significantly associated with phenol color indicating the importance of this region. These SNPs explained 15.1–18.6% phenotypic variation. Out of 9 SNPs associated with phenol color, 6 SNPs encoded 5 proteins (Table 3). The transcript profile of nucleoredoxin 1-2 (NRX1) gene encoded by SNP AX-94738314 associated with phenol color showed highest expression (FPKM-272.53) in grain at Zadok 72 stage (early milk). Protective effect of NRX1 boosted the H_2_O_2_ detoxification capacity of catalase, thereby protecting the plant cell from oxidative stress (Kneeshaw et al., 2017). Significant SNPs associated with grain texture (phenotype scored as hard or soft), encoded four proteins (Table 3). Most of the proteins identified in this study for grain traits were similar to proteins reported by Arora et al. (2017) for grain related traits.

The genetic architecture of the quantitative traits is complex as controlled by many loci with small effect. Several significant markers mined for six complex traits in this study were co-localized with the previously reported QTL regions (Table 2). Flowering is controlled by a complex network of genes integrating vernalization response genes (*Vrn*) on chromosomes 5A (*Vrn-A1*and *Vrn-A2*) and 7BS (*Vrn-A3*), photoperiod response gene on chromosome 2, and earliness genes on chromosomes 1A and 3A (Fowler and Laudencia-chingcuanco, 2016). The MTAs for DTH were detected on three chromosomes 4A, 5A, and 7D. MTA AX-95187165 (89.02 cM) identified for DTH on chromosome 5A was mapped at nearly same position of the functional gene *Vrn-A1* (90 cM) indicating its role for determining DTH (Sukumaran et al., 2014). Another putative candidate, AX-94542441 (chromosome 4A) associated with DTH is an ortholog of shikimate kinase-like protein. Likewise in rice (*Oryza sativa*) two shikimate kinase isoforms OsSK1 and OsSK3 accumulate to high levels during the heading stage of panicle development and involved in floral organ development (Kasai et al., 2005). The most significant association for DTH was found on chromosome 7D stable in four environments (E1–E4). On similar lines, Lopes et al. (2013) also reported a significant QTL for DTH on chromosome 7D, associated for more than 30% of DTH variation but at different position.

The SNPs significantly associated with DTM were identified on chromosomes 3B, 5A, 5B, and 6A corresponding to the earlier reported genomic regions for DTM on chromosome 3B (Sukumaran et al., 2014, 2018; Okechukwu, 2017), 5A (Gahlaut et al., 2017), 5B (Gahlaut et al., 2017; Zou et al., 2017), and 6A (Sukumaran et al., 2014). The significant QTL for DTM harboring three SNPs were observed on chromosome 3B (61.38, 84.9, and 85.27 cM) were closely co-localized with the previously identified MTAs for DTM, indicating that these QTLs were stable and could be detected in different environments. Another noteworthy region, associated with DTM was identified on chromosome 2A (179.61 cM) consistent in three environments encoding BTB/POZ domain and ankyrin repeat-containing protein which plays key role in plant growth and development stages (Sharma and Pandey, 2016). Moreover, chromosome 2A associated with DTM also encompasses a region (83.23 cM) governing multiple traits (PH and SL) thereby representing the correlation of these traits in diverse panel in agreement with the previous reports (Kamani et al., 2017; Zhai et al., 2016).

For PH, as many as six MTAs were identified on chromosome 1B, 2A, 2B, 5D (2), and 7A. The genomic region of MTA (AX-94941145) identified at 29.9 cM on chromosome 7A falls in the region of the reduced height gene *Rht22* (*Xgwm471-*29.5 cM, *Xgwm350-*20.1 cM) (Peng et al., 2011). Similarly, the MTA identified for PH on chromosome 2B (104.59 cM) and 5D (1.58 cM) found in proximity to the reduced height genes *Rht4* (*Xwmc317-*106 cM) (Ellis et al., 2005) and *Rht23* (*Xgdm63-*4.7 cM, *Xbarc110-*11.1 cM) (Chen et al., 2015). We also observed an MTA on chromosome 2A where *Rht7* gene had already been reported but at different map position. For two correlated traits PH and DTM (Mohibullah et al., 2012), a significant MTA (AX-94656878) was detected on chromosome 2A annotated *bZIP* transcription factor. In plants, these factors regulate genes in response to abiotic stress, seed maturation, flower development and pathogen defense (Jakoby et al., 2002). Similarly, several studies reported a moderate, but significant correlation between heading time and PH (Sultana et al., 2002; Mohibullah et al., 2012). The MTA AX-94941145 identified on chromosome 7A annotated probable LRR receptor-like serine/threonine-protein kinase At3g47570. Further, we investigated the possibilities of semi dwarfing genes on chromosome 4B and 4D which is present in Indian cultivars (Sheoran et al., 2013) but could not detect any MTA linked to these genes (*Rht-B1b* and *Rht-D1b*) suggesting either these genes were eliminated during filtering or may not reach significant threshold level. Similarly, Ain et al. (2015) did not find any MTA for semi dwarfing genes on chromosome 4B and 4D employing iSelect 90K SNP chip. Several MTAs for PH have been reported previously on chromosomes 2A (Ain et al., 2015; Mengistu et al., 2016), 2B (Zanke et al., 2014; Ain et al., 2015; Gao et al., 2015) and 7A (Gao et al., 2015;Soriano et al., 2017).

Wheat domestication genes *Q, compactum* (*C*), *sphaerococcum* (*S1*) related to spike morphology have been detected on chromosomes 5A, 2D, and 3D, respectively (Johnson et al., 2008). In the present study, eight loci were detected on chromosomes 1B, 2A, 3A, 3B, 3D, 5A, 7A, and 7B for SL which were partially consistent with those of Zhou et al. (2017), who reported QTLs for SL on chromosome 3A, 3B, 5A and with those of Ma et al. (2007), who reported genomic regions for SL on chromosome 1B and 7A. These results indicated that multiple loci having unequal effects can influence the variations in the SL. It is interesting to note that PH shared common significant loci (83.23 cM) with SL showing a high correlation between these traits in concurrence with the previous results (Sukumaran et al., 2014). Besides MTAs on chromosome 1B (discussed earlier), another SL associated SNP AX-94722223 (chromosome 5A) harboring actin related protein (ARPC3) which has been known to play a key regulator of cytoskeleton dynamics controlling multiple developmental processes in a variety of tissues and cell types (Qi et al., 2017). We expected genes contributing to variation in SL to be most strongly expressed within the different growth stages of spike. In fact, 5 putative candidate genes identified for SL showed expression FPKM >5 in spike tissue depicting highest expression (FPKM 143.65) at growth stage 65 (Zadok’s Scale), corroborating its causal effect.

Awns were reported superior to the flag leaves on a cellular and physiological level during the grain filling period contributing 40–80% of the photosynthetic assimilates accumulated in the wheat grain (Li et al., 2006). Most significant MTAs for Awn_L were reported on several chromosomes *viz*., 1A, 1B, 2B, 2D, 3B, 4A, 5B, 6D, and 7B (Wu et al., 2016; Liu Y. et al., 2018). However, some of the chromosomal regions associated with Awn_L for instance 1.72 cM (7B) and 85.27 cM (3B) were detected for both Awn_L and SL. This study further corroborated the result of Echeverry-Solarte et al. (2015), who reported a novel QTL for Awn_L on chromosome 7B harboring two consistent loci associated to supernumerary spikelet (SS) and putative QTLs for PH, DTH, DTM making it an important loci for future studies. In the present investigation, cell elongation protein *DIMINUTO* predicted for Awn_L associated SNP locus (AX-95025537 and AX-95012310) on chromosome 7B (Supplementary Table S8) have been implicated in regulating cell elongation (Takahashi et al., 1995). The genomic regions that contributed to Leaf_L were found associated with chromosomes 4A and 5A with phenotypic variations ranging from 15.5 to 15.7%. In earlier reports several chromosomal regions *viz*., 2B, 3A, 4A, 4B, and 5A were detected for flag Leaf_L (Jia et al., 2013; Wu et al., 2016). Positive and significant correlation between flag Leaf_L and SL revealed their role in increasing yield (Wu et al., 2016).

Mining of superior/favorable alleles is essential for improving the complicated earliness trait in wheat using marker assisted selection. In recent years, association mapping has been widely used in exploring the elite alleles of many agronomic traits such as yield related traits (Sun et al., 2017), heading days and PH (Ain et al., 2015; Ogbonnaya et al., 2017) and water soluble carbohydrates (Dong et al., 2016) in wheat. In the present study, the phenotypic effect value of the favorable alleles of DTH, PH, and SL was evaluated and inferred to have positive effect on the respective traits. The candidate genes and the SNPs linked with the economic important traits identified in this study could help in designing new strategies to hoard superior alleles for these key traits in future marker based breeding. Some novel regions identified in the present investigation could have been previously detected, but comparison of the positions of the SNPs linked to the respective traits was not possible due to the limitations of the various marker system used in different studies.

This study identified 146 MTAs for 23 agro-morphological traits (Supplementary Table S9), and putative candidate genes using the recently released genome sequence by IWGSC RefSeq v1.0 (Appels et al., 2018). MTAs specific to less explored traits such as awn length and glume pubescence were targeted for visualizing future needs of breeders in developing efficient and resilient wheat varieties. The chromosomal region controlling multiple traits were also identified which should pave the way for selection and may prove effective for pyramiding favorable alleles. Here we discovered novel candidate genomic regions together with previously reported genes which require further validation and testing in the wheat germplasm. Therefore, the significant MTAs identified having known candidate genes are being subjected to conversion as Kompetitive Allele Specific PCR (KASP) markers that can be efficiently used to transfer alleles into elite wheat genotypes (Rasheed et al., 2016). These useful genomic resources and PCR based markers (KASP markers) could be utilized for introgression of traits through marker assisted selection (MAS). These will strongly enhance systematic study of the genetics, comparative genomics and evolution of wheat, and will expedite isolation and characterization of genes controlling agronomically important traits, such as yield and abiotic stress.

## Author Contributions

RT and DiK conceived the theme of the study. SS, DeK, NR, RuS, SusP, SJ, MI, AJ, NK, UA, and SurP did the computational analysis. SS, SJ, MI, RaS, PS, RT, and DiK drafted the manuscript. DeK, NR, RuS, CS, and AG did the phenotyping. NR and DeK contributed in wet lab work. SJ, MI, DiK, AR, GS, and RT edited the manuscript. All authors read and approved the final manuscript.

## Conflict of Interest Statement

The authors declare that the research was conducted in the absence of any commercial or financial relationships that could be construed as a potential conflict of interest.

## Footnotes

1 www.fao.org/faostat/en

2 www.wheatatlas.org/varieties

3 http://www.upov.int/edocs/tgdocs/en/tg003.pdf

4 http://wheatatlas.org/megaenvironments

5 http://plantauthority.gov.in/pdf/PVJFebruary2011.pdf

6 http://www.sas.com

7 http://www.cerealsdb.uk.net/cerealgenomics/CerealsDB/Excel/35K_array/Supplementary_file_3.xlsx

8 http://zzz.bwh.harvard.edu/plink/

9 http://www.wheatgenome.org/

10 http://plants.ensembl.org/Triticum_aestivum

11 https://wheat.pw.usda.gov/WheatExp/

12 http://pngu.mgh.harvard.edu/purcell/plink

13 https://patents.justia.com/patent/6624343
