# Supplementary material for: Uncovering Genomic Regions Associated With 36 Agro-Morphological Traits in Indian Spring Wheat Using GWAS
Source: Front Plant Sci. 2019 Apr 25;10:527. doi: 10.3389/fpls.2019.00527 (PMC6511880; doi:10.3389/fpls.2019.00527)
Supplement: Supplementary file 1 [file Data_Sheet_1.docx]

***Supplementary file S1***

**List of 36 agro-morphological traits and their corresponding method for observation**

**1. Coleoptile Color (anthocyanin)/ C_Col**

About 50 grains were set out on moist filter paper in a petri dish for germination in laboratory. After coleoptile had reached a length of about 1 cm in darkness, petri dish were placed in artificial light or sunlight at 15,000 lux continuously for 3-4 days at 15-20◦C. Coleoptile color was rated as **Absent (Green) and Purple.**

**2. Plant Growth Habit/ PGH**

Different cultivars in their early stages of growth marked difference in their growth habit. The character is recorded at tillering stage between 30-45 days after sowing. When a plant grows vertically or nearly so making a right angle, characters is classified as **Erect. On the basis of culm angle it is classified as Semi-erect, Intermediate, Semi-spreading, and Spreading.**

**3. Foliage Colour/ Fol_Col**

This character is observed at the time of flowering. On the basis of intensity of the colors of flag leaves, varieties are characterized into three categories i.e. **Dark green, Green and Light or Pale green.**

**4. Flag leaf: Anthocyanin coloration of auricle/ Aur_Col**

Some varieties have pigmented auricals while others do not have pigments on them. On the basis of colour intensity, this character is classified as strong (**dark purple**), weak (**purple**) and colorless (**green**). The colour intensity is affected by exposure of leaf to sunlight and should be observed at the boot stage, since at later stage, the intensity of colour starts fading.

**5. Flag leaf : Hairs on Auricle/ Aur_Pub**

Hair intensity on auricle at boot stage vary in different genotypes and can be classified as **absent (no hairs), Medium/ Moderate (few hairs), Strong (tuft of hairs).**

**6. Flag leaf attitude/Leaf_Att**

Based on curvature of flag leaf lamina at inflorescence stage it is classified in to **Erect, Semi-erect, Semi drooping, and Drooping.**

**7. Ear: Time of emergence (first spikelet visible on 50% of ears)/DTH**

It is counted as number of days taken from sowing to the day when 50% plants show ear emergence.

**8. Flag Leaf: Waxiness of sheath/ Wax_LS;9. Flag Leaf: Waxiness of blade/Wax_LB;10. Ear: Waxiness/Wax_Ear;11. Peduncle: Waxiness of neck/Wax_Ped**

Some varieties developed whitish powder like material (wax) on plant parts and its presence is classified as waxy and when absent , it is glaucous or non waxy. Based on intensity of wax present on plants parts it is classified in to **Absent, Weak, Medium, Strong and Very strong.**  These characters are observed at the time of flowering because at Lateral stages waxiness starts disappears. It is observed on ear, leaf blade, leaf sheath and peduncle.

**12. Flag leaf: Length/Leaf_L**

Average flag leaf length of two plants per genotypes measured in centimeters.

**13. Flag leaf: Width/Leaf_Br**

Flag leaf length measured in centimeters at the point where lamina or blade is broadest and averaged. It is characterized as **Narrow (<1cm), Medium (1.01-1.7cm) and Broad (>1.7cm).**

**14. Plant height (excluding awns/ scurs)/PH**

This character is measured at the time of maturity. It is measured from base of the plant to the tip of spike excluding awns in centimeters.

**15. Ear: Shape in profile/Ear­­_S**

It is observed at the time of maturity, can be used as varietal character and ear shape in profile can be characterized **Tapering, Parallel, Club shaped**. The ears which tapers from the side or face view are called tapering. Ear with equal sides are called as parallel. Some parallel ears show clubbing of spikelet at the top of the ear is called as clubbed whereas ears which are wider in the middle and taper at both ends are called as fusiform.

**16. Ear: Density/Ear_D**

Density can be observed at maturity by relative length of the rachis segments. In dense ear, rachis segments are relatively short and spikelets are compact. In lax ears, the spikelets are apart and rachis segments are easily visible between the spikelets. Based on visibility of rachis segments, ear density can be characterized into five groups i.e. **Very lax, Lax, Medium, Dense, Very dense**.

**17. Spike Length/ SL**

It is measured from the tip of apical spikelet to the base or collar of the ear excluding awns in centimeters.

**18. Awns or scurs: Presence/Awn_P**

At maturity, on the basis of presence and absence of awn genotypes were classified as aweless (awn **Absent), Scur present (small projections), and awned (Awns present).**

**19. Awns: Length/Awn_L**

Awn length is measured in centimeters from 1/3 middle part of ear at maturity and averaged.

**20. Awns: Color/Awn_Col**

Awn color is observed at the time of maturity and classified as **White (pale), Light brown, Dark brown, Black.**

**21. Awn: Attitude/Awn_Att**

It is observed at late dough stage. Depending upon the angle of awns to rachis, it is characterized as **Appressed (almost attached to ears), Medium, Spreading**.

**22. Outer glume: Pubescence/Glu_Pub**

Ears differ in the intensity of hairs present on glume and are classified into absent (smooth), medium, strong, and very strong.

**23. Ear: Colour/Ear_Col**

Ear color is recorded at the time of maturity and can be characterized as **White, Light brown, Brown.**

**24. Lower glume: Shoulder width (spikelets in mid- third of ear)/Sh_Wid**

It refers to the upper part /edge of the wing. When upper part is not extending into the wings, it is called absent or very narrow, slightly extension is termed as narrow, when it is 1/3 of the base then termed as medium and those which are broader at the top are classified as broad and very broad viz. **Absent, Narrow, Medium, Broad, Very broad.**

**25. Lower glume: Shoulder shape /Shl_Sh**

It refers to the upper part (edge) of the broad wing on the opposite side from the beak. When it slopes down the beak, it is termed as sloping and when it makes a curve, it is called round. If the shoulder is raised upward then the shape is termed as elevated. Shoulder is at right angle to the beak is called as square and in case the shoulder raised making an acute angle with beak is called indented viz.Sloping, Round, Straight, Elevated, Indented.

**26. Lower glume: Beak length /Beak_L**

Length of the beak is measured in millimeters from its tip down the keel (folded edge) to the point where it arises from the glume. If the beak is absent or the length is under 1mm, it is classified as absent or very short. Based on length, it is categorized in to short (< 1 -3), medium (<3-5), long (<5-7) and very long (>7).

**27. Lower glume: Beak shape (as for 25)/Beak_Sh**

The beak of outer glume may be straight or curved. On the basis of the degree of curvature shape is classified in to **straight** or weak, moderately curved and strongly curved.

**28. Spike (peduncle) attitude (at the time of maturity)/Ped_Att**

This character is observed at the time of maturity. Top of the peduncle is not straight always but show a different shape and classified as **Straight, Bent, and Crooked (wavy).**

**29. Grain: Coloration with phenol/Grn_Ph**

The phenol test is conducted soaking seeds in distilled water for 16 -20 hours, then the water is drained off and grains are set out on filter paper in a petridish with crease downwards. Freshly prepared 1% phenol solution is added, sufficient to cover ¾ of grain. After 4 hours, phenol solution is drained off and seeds are dried on filter paper. The enzyme phenol oxidase is responsible for the development of colour on wheat seeds which is due to enzymatic action of tyrosinase present in the seed coat. Phenol colouration is classified into five categories depending upon the intensity of colour i.e. , none or nil , light brown ( light ) , brown ( medium ) , dark brown ( dark ) and very dark or black .

**30. Grain: Color/Grn_Col**

Based on seed coat color, grain color is classified as **White, Amber, and Red**

**31. Grain: Shape/Grn_Sh**

Shape of grain is classified as **Round** (when grain appears spherical), **Ovate** (grain is small in length with narrow ends), **Oblong** (slightly longer with both sides blunt), and **Elliptical** (gain is elongated).

**32. Grain: Germ width/Germ_Wid**

At the base of grain small wrinkled patch which covers the entire embryo within, is called germ and the character is used in cultivar identification. It is classified into three categories i.e. wide (when covers almost whole width of grain), medium (2/3 is covered) and narrow. (1/3 of grain is covered).

**33. Brush hair: Length/Brush_L**

On the basis of length brush hairs are grouped in to prominent (when hairs are long and visible with naked eye), medium small hairs present and not prominent when hairs are either absent or very short and cannot be seen by naked eye. This observation is recorded with the help of magnifying glass and based on majority of grains in the sample.

**34. Seed: Size (weight of 1000 grains)/Grn_Size**

Based on thousand grain weight and visual observation. The grain are classified as **Small (<35g), Medium (35.1-40g), Large (40.1-45g), and Very large (>45g).**

**35. Grain: Hardness/Grn_Tex**

This can be observed by cutting few grains with blade and observing the amount of flintiness in cross section. This can be classified as **Hard, Semi hard and Soft.**

**36. Days to Maturity/ DTM**

It is counted as number of days taken from sowing to the day when 50% plants showed their peduncle dried due to physiological maturity.
