# Supplementary figures and images for: Uncovering Genomic Regions Associated With 36 Agro-Morphological Traits in Indian Spring Wheat Using GWAS
Source: Front Plant Sci. 2019 Apr 25;10:527. doi: 10.3389/fpls.2019.00527 (PMC6511880; doi:10.3389/fpls.2019.00527)

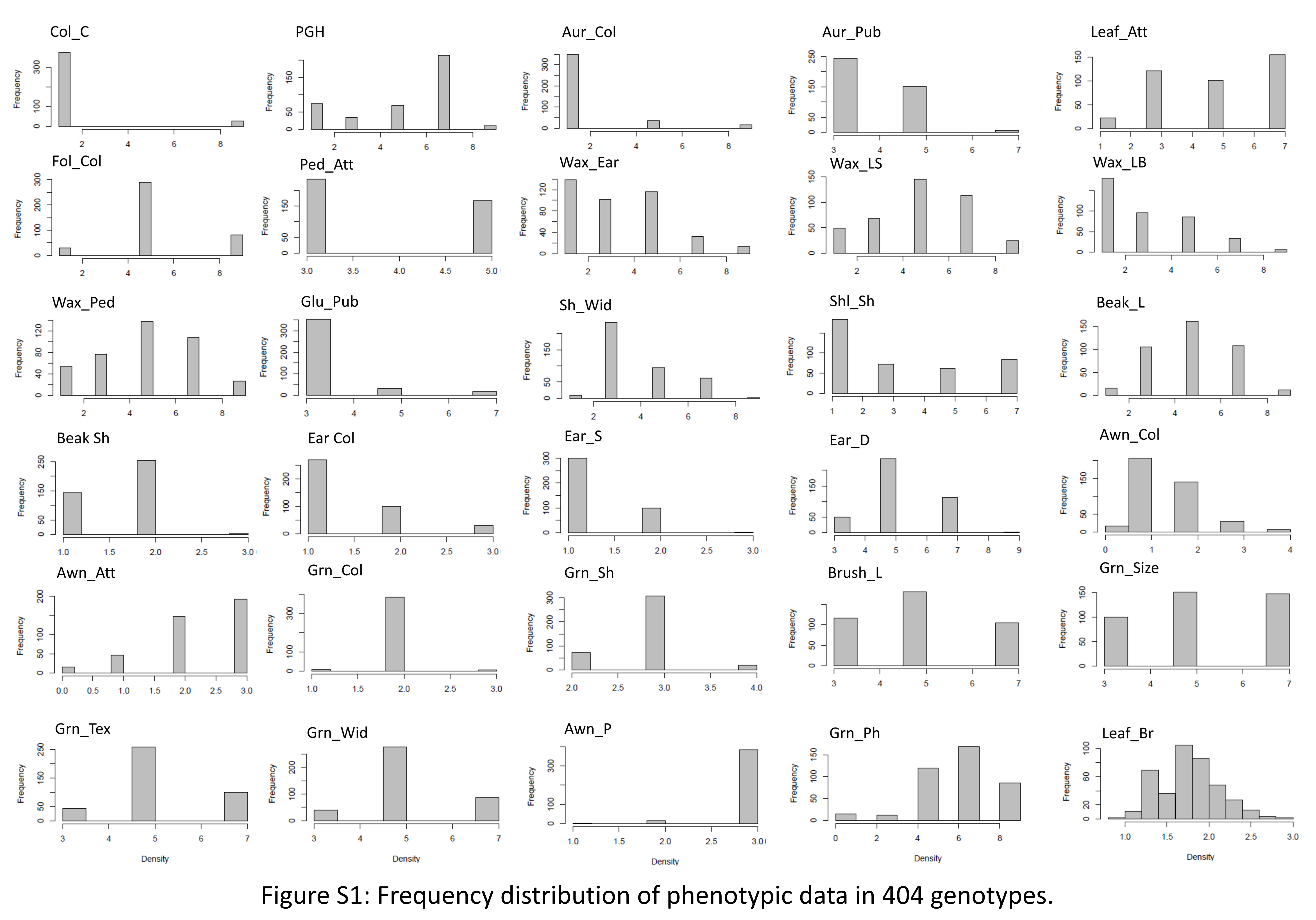

Supplement: Supplementary file 2 [file Image_1.TIF]

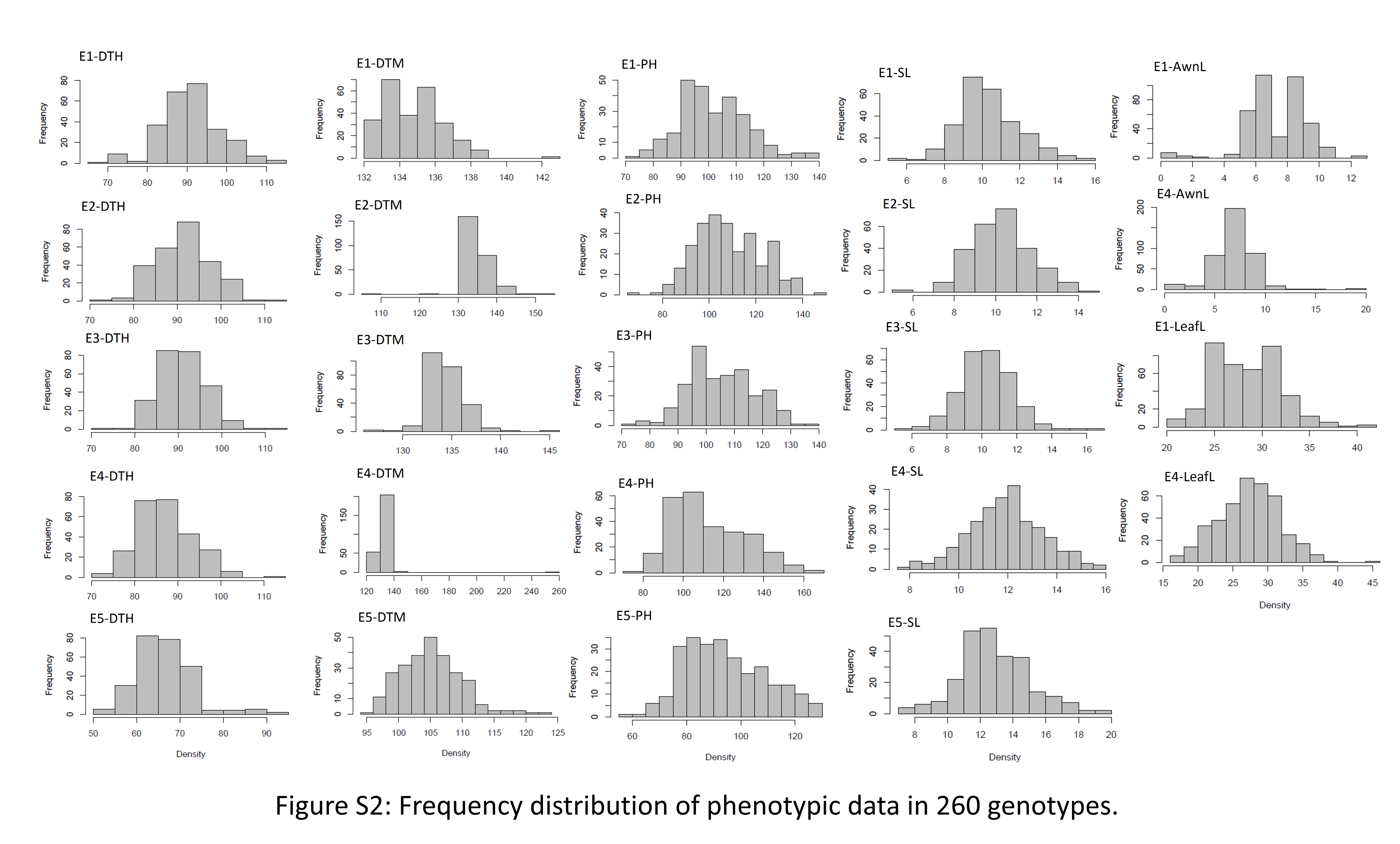

Supplement: Supplementary file 3 [file Image_2.TIF]

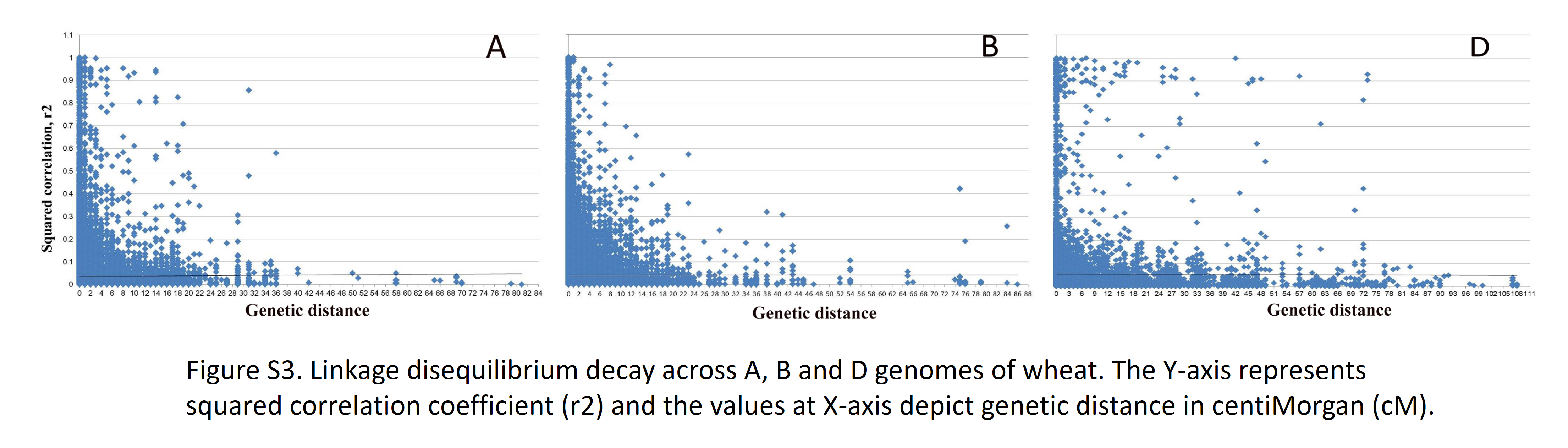

Supplement: Supplementary file 4 [file Image_3.TIF]
