# Supplementary material for: Uncovering Genomic Regions Associated With 36 Agro-Morphological Traits in Indian Spring Wheat Using GWAS
Source: Front Plant Sci. 2019 Apr 25;10:527. doi: 10.3389/fpls.2019.00527 (PMC6511880; doi:10.3389/fpls.2019.00527)
Supplement: Supplementary file 5 [file Image_4.pdf]

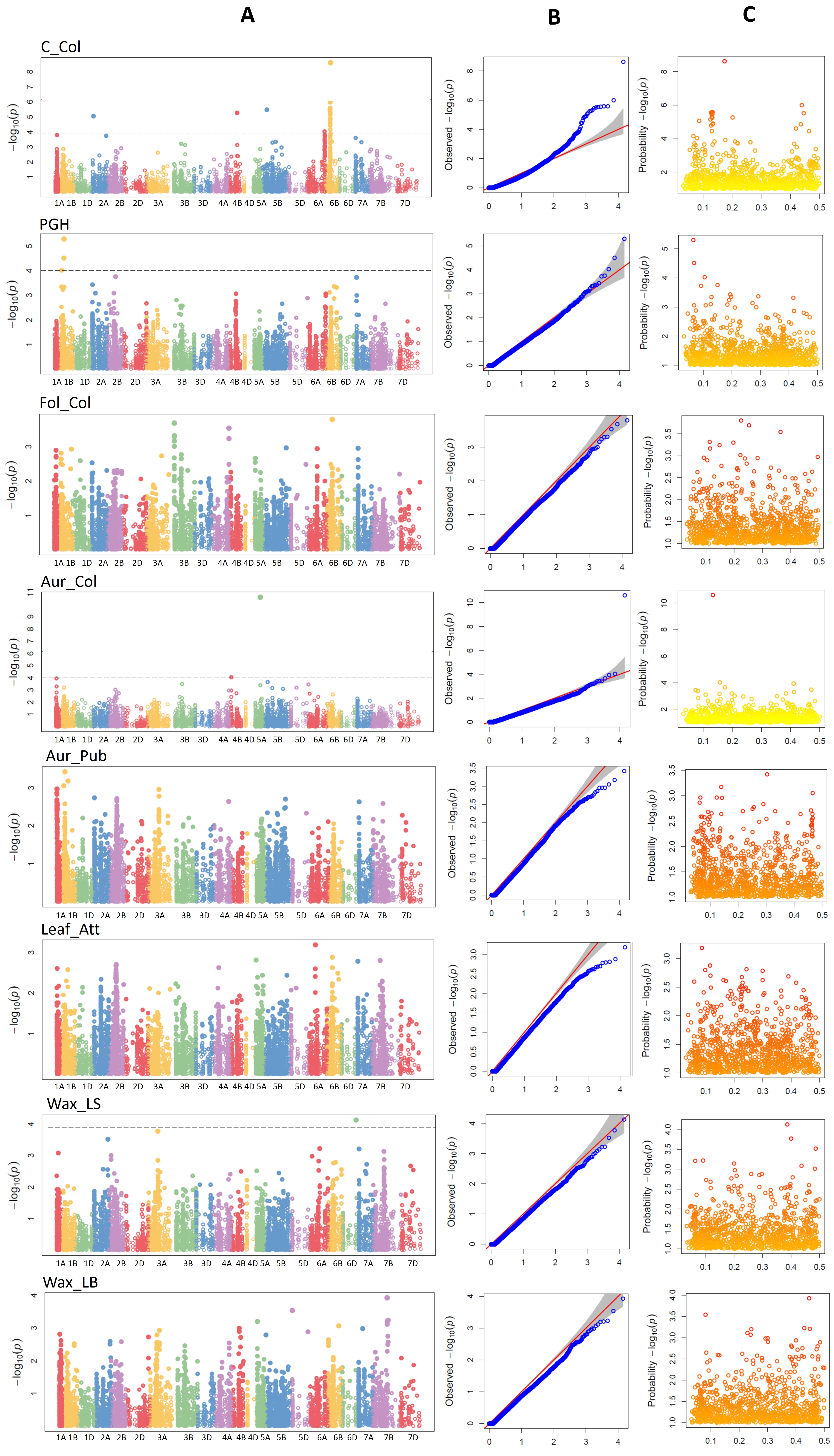
A
B
C
C_Col
PGH
Fol_Col
Aur_Col
Aur_Pub
Leaf_Att
Wax_LS
Wax_LB
$-\log_{10}(p)$
Observed $-\log_{10}(p)$
Probability $-\log_{10}(p)$
1A 1B 1D 2A 2B 2D 3A 3B 3D 4A 4B 4D 5A 5B 5D 6A 6B 6D 7A 7B 7D

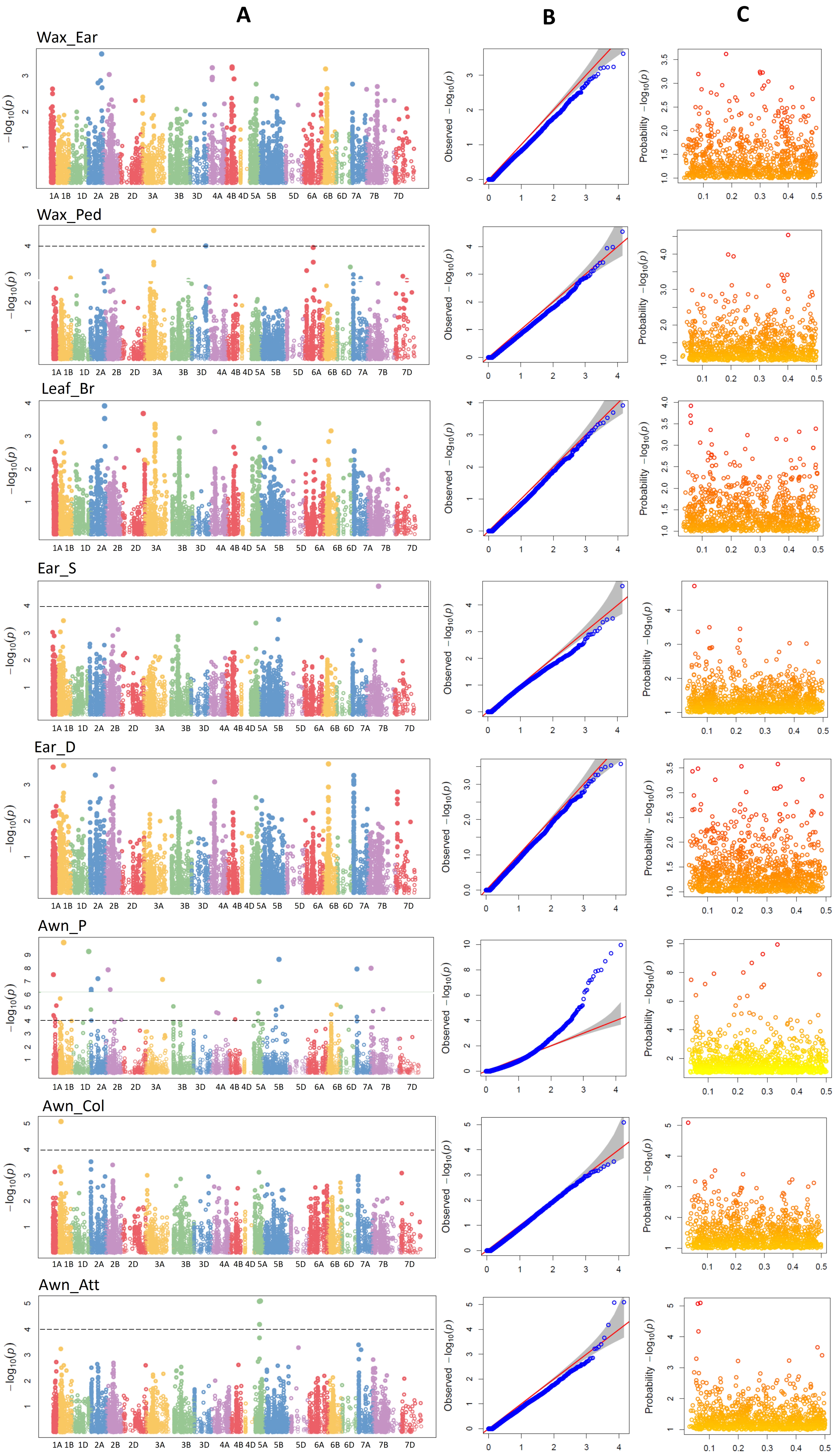
A
B
C
Wax_Ear
Wax_Ped
Leaf_Br
Ear_S
Ear_D
Awn_P
Awn_Col
Awn_Att
−log10(p)
Observed −log10(p)
Probability −log10(p)
1A 1B 1D 2A 2B 2D 3A 3B 3D 4A 4B 4D 5A 5B 5D 6A 6B 6D 7A 7B 7D

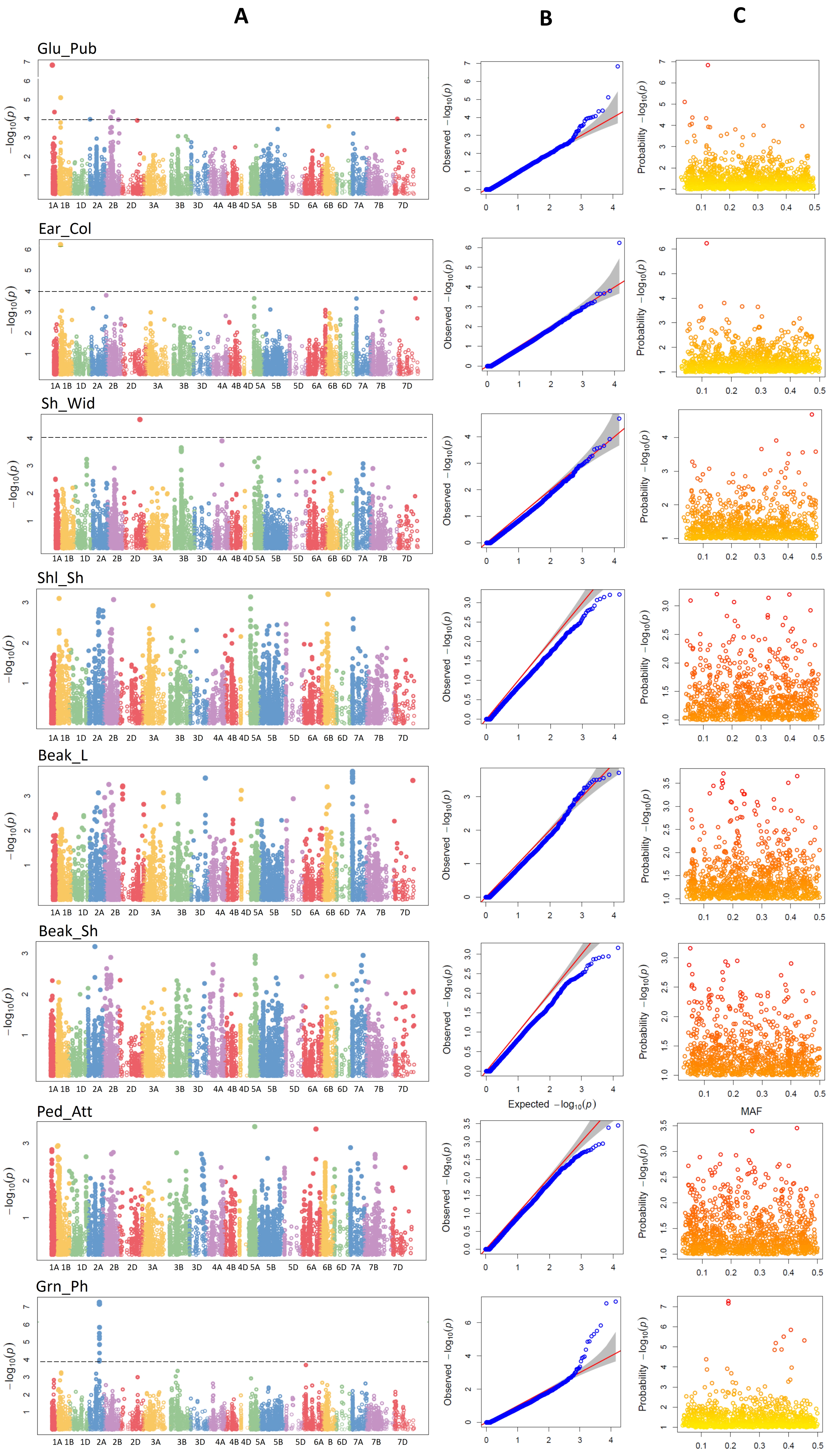
A
B
C
Glu_Pub
Ear_Col
Sh_Wid
Shl_Sh
Beak_L
Beak_Sh
Ped_Att
Grn_Ph
−log10(p)
Observed −log10(p)
Expected −log10(p)
Probability −log10(p)
MAF
1A 1B 1D 2A 2B 2D 3A 3B 3D 4A 4B 4D 5A 5B 5D 6A 6B 6D 7A 7B 7D

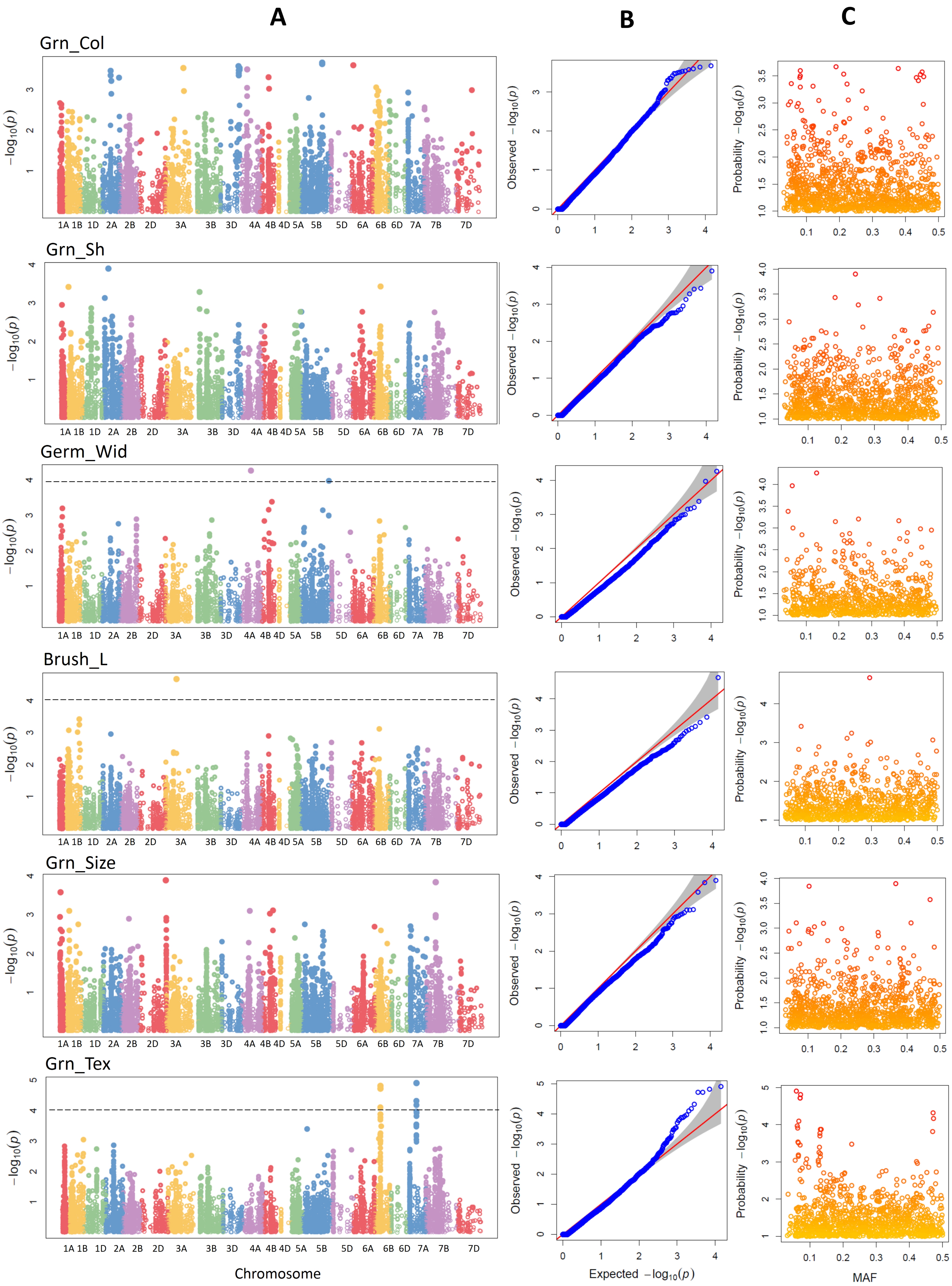

Figure S4: Manhattan, Q-Q and Minor allele frequency (MAF) plot for Agro-morphological traits.
