# Supplementary material for: Uncovering Genomic Regions Associated With 36 Agro-Morphological Traits in Indian Spring Wheat Using GWAS
Source: Front Plant Sci. 2019 Apr 25;10:527. doi: 10.3389/fpls.2019.00527 (PMC6511880; doi:10.3389/fpls.2019.00527)
Supplement: Supplementary file 6 [file Image_5.pdf]

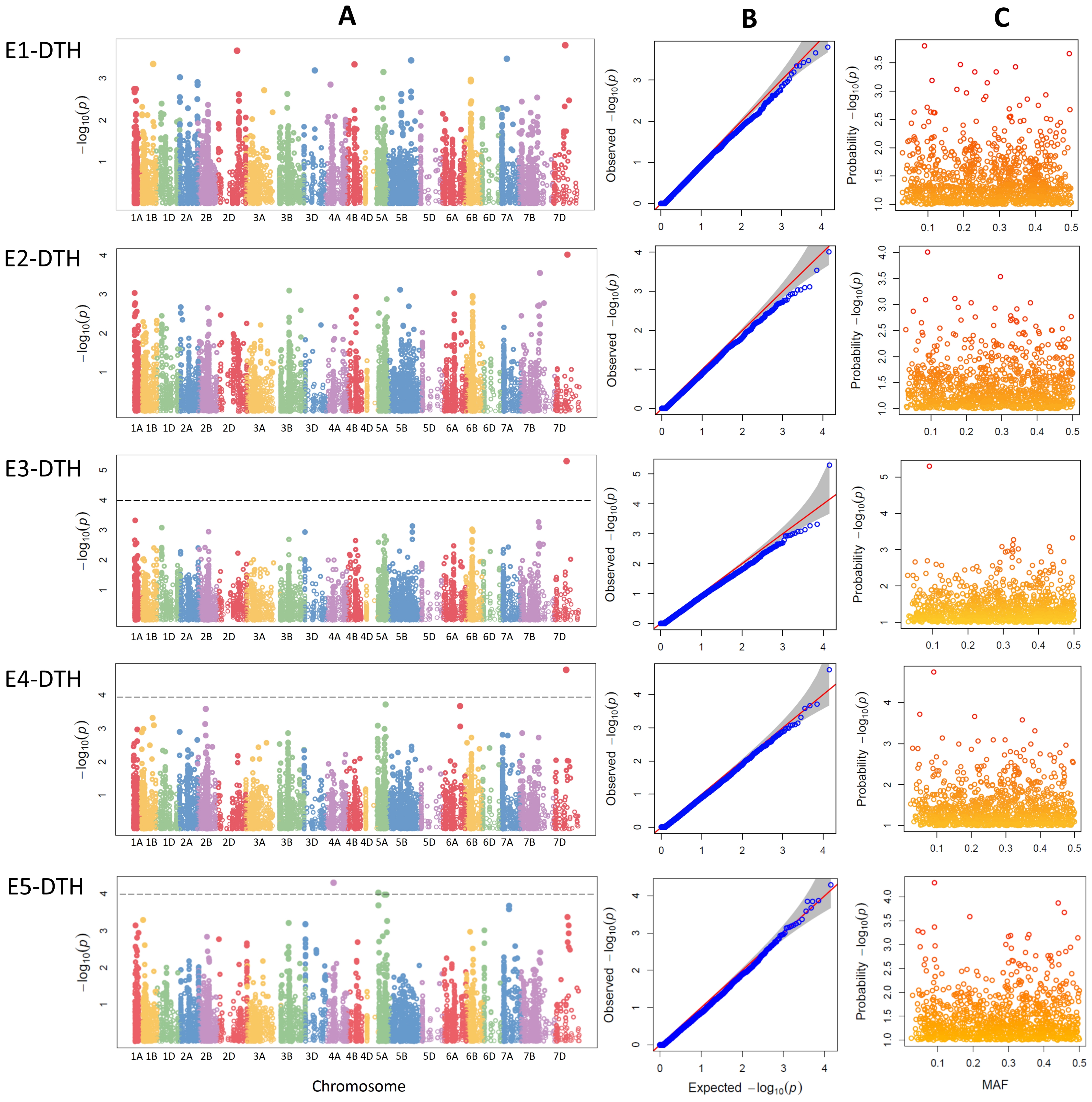

Figure S5-1:(A) Manhattan plot, (B) Q-Q plot and (C) MAF for Days to Heading (DTH)

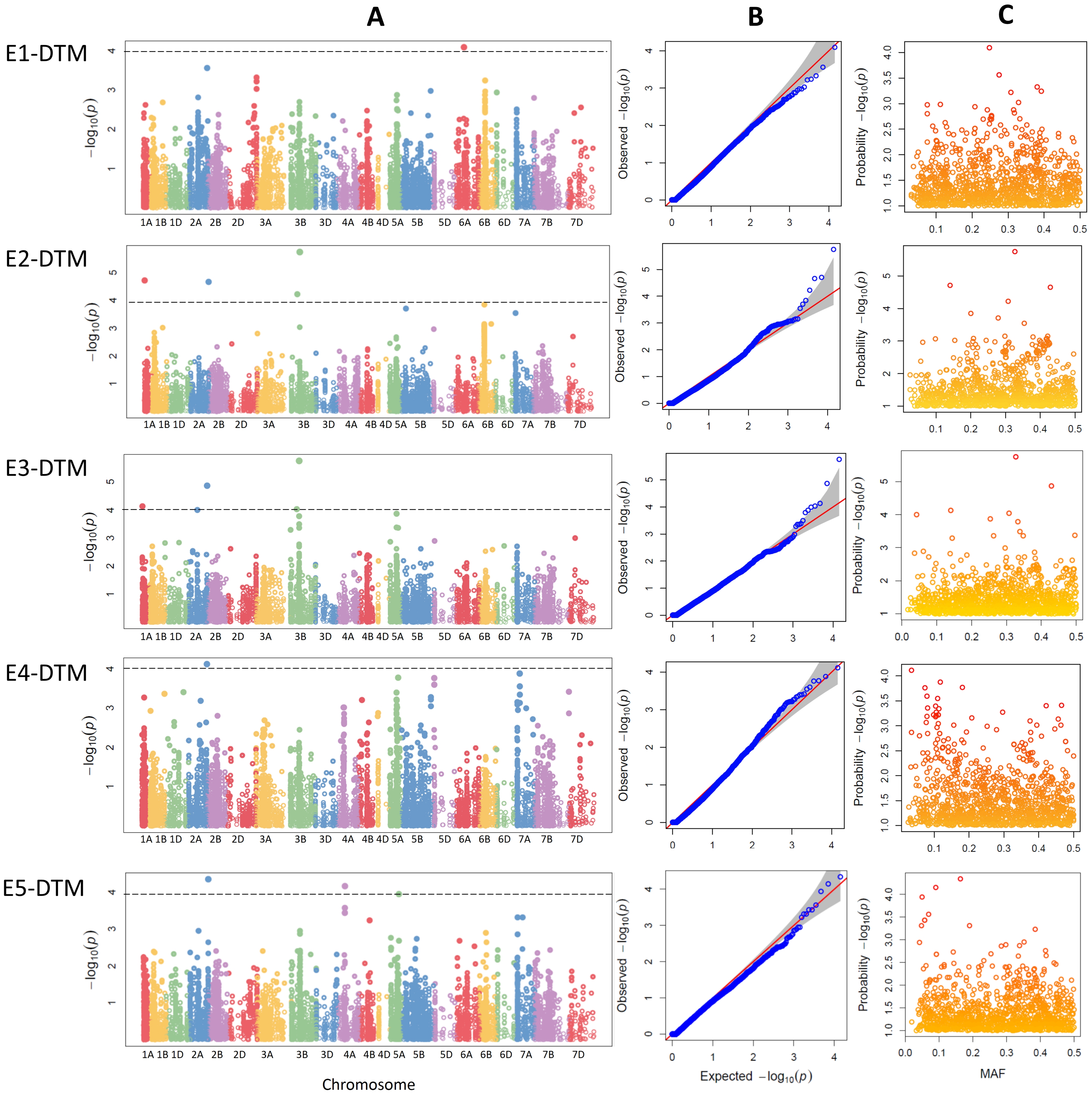

Figure S5-2: (A) Manhattan plot, (B) Q-Q plot and (C) MAF for Days to Maturity (DTM)

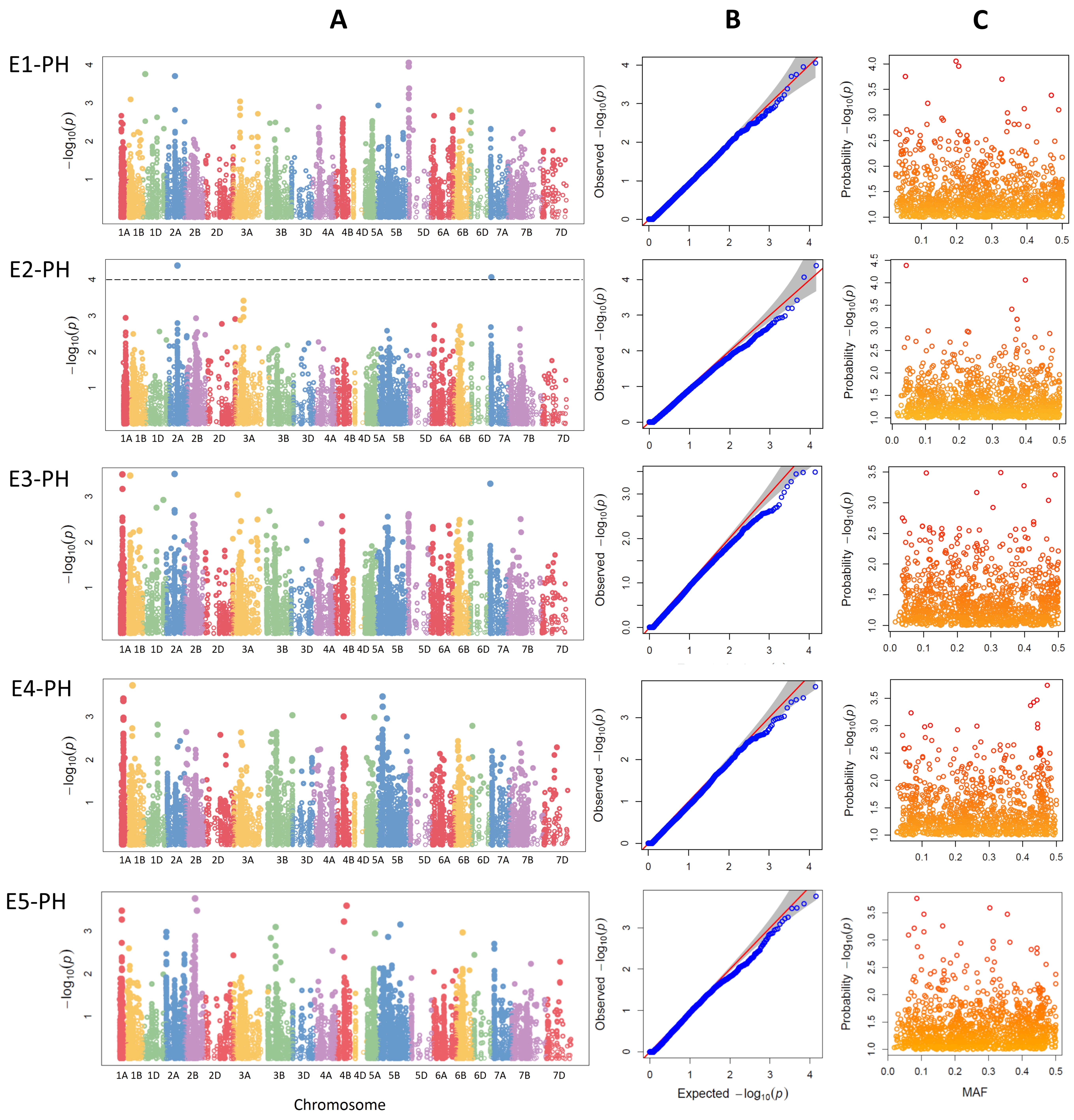

Figure S5-3:(A) Manhattan plot, (B) Q-Q plot and (C) MAF for Plant Height (PH)

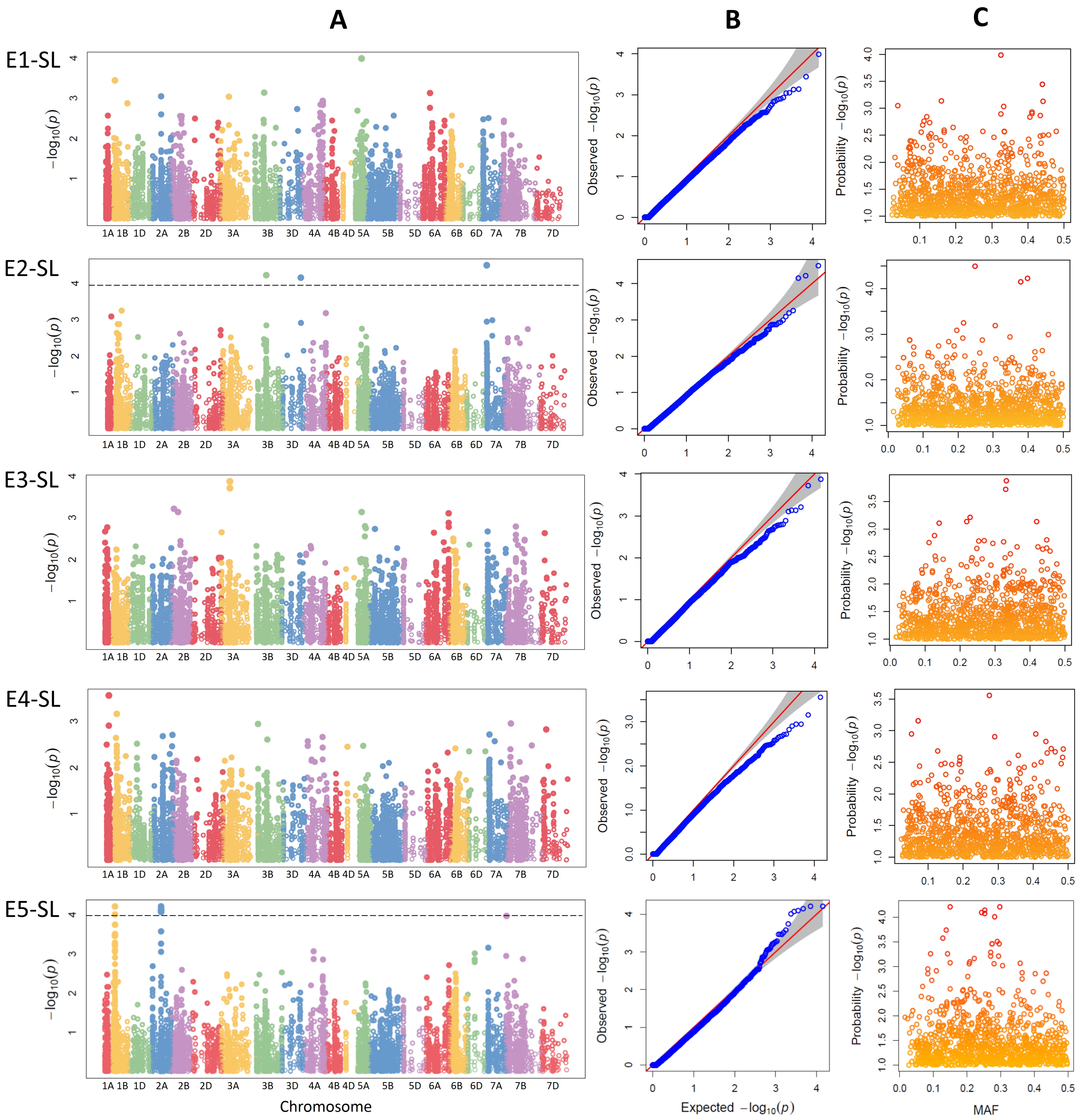

Figure S5-4: (A) Manhattan plot, (B) Q-Q plot and (C) MAF for Spike Length (SL)

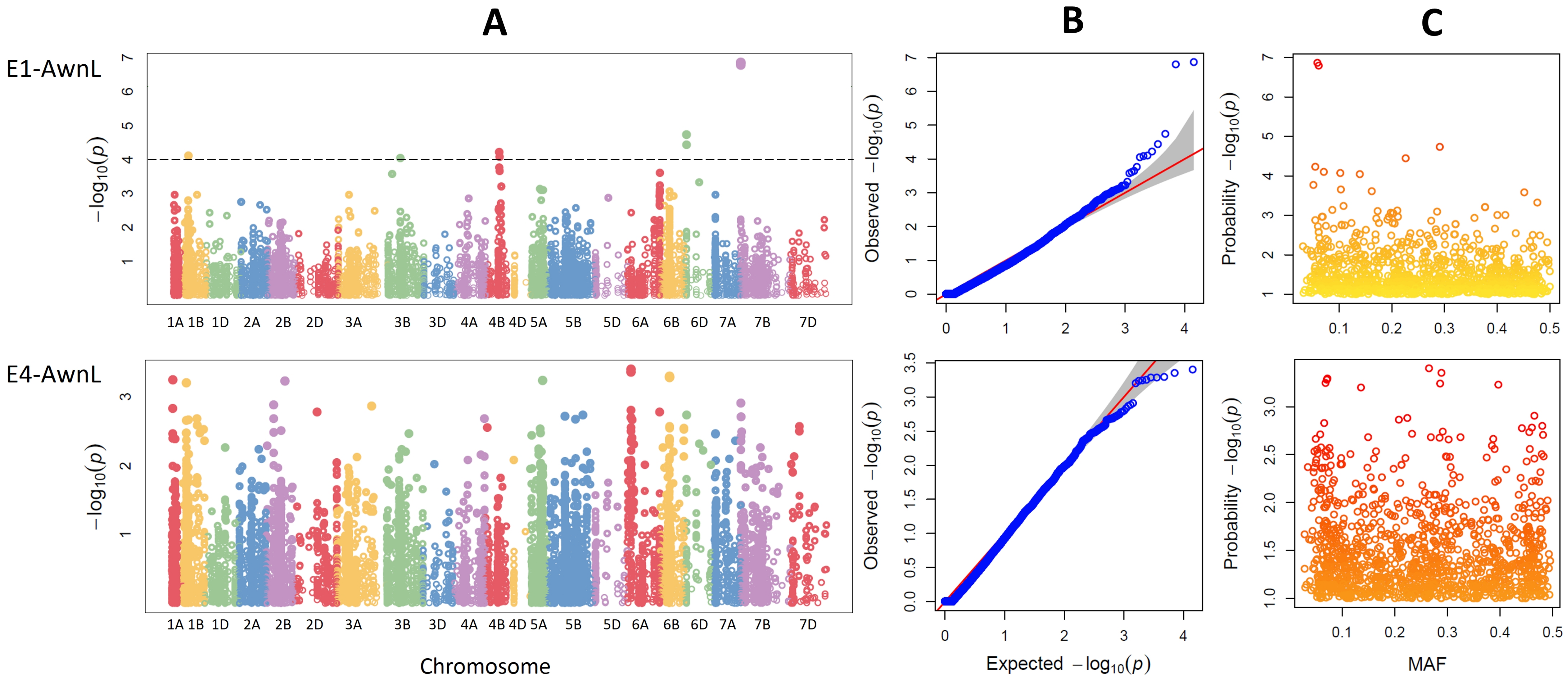

Figure S5-5:(A) Manhattan plot, (B) Q-Q plot and (C) MAF for Awn Length (AwnL)

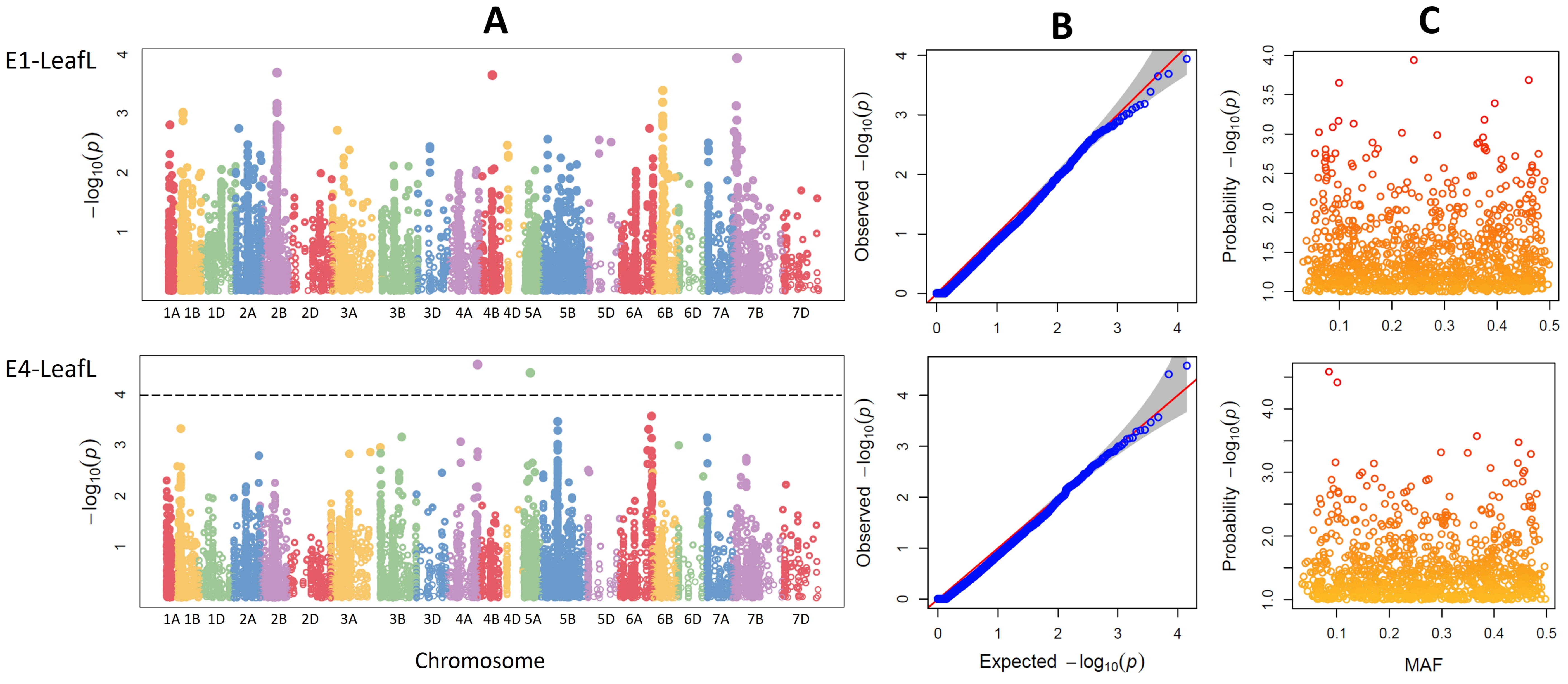

Figure S5-6:(A) Manhattan plot, (B) Q-Q plot and (C) MAF for Leaf Length (LeafL)
