# Supplementary material for: Uncovering Genomic Regions Associated With 36 Agro-Morphological Traits in Indian Spring Wheat Using GWAS
Source: Front Plant Sci. 2019 Apr 25;10:527. doi: 10.3389/fpls.2019.00527 (PMC6511880; doi:10.3389/fpls.2019.00527)
Supplement: Supplementary file 9 [file Table_3.docx]

**Table S3: Basic statistics of the phenotypic variations observed for six quantitative traits**

| Trait | Env. | Mean | Std.Dev. | Min. | Max. | Mode | Range | CV |
| --- | --- | --- | --- | --- | --- | --- | --- | --- |
| DTH (days) | **E1** | 91.54 | 7.41 | 68.00 | 113.00 | 90.00 | 45.00 | 8.10 |
|  | **E2** | 92.08 | 6.14 | 74.00 | 113.00 | 94.00 | 39.00 | 6.60 |
|  | **E3** | 91.12 | 5.24 | 71.60 | 113.00 | 88.00 | 42.00 | 6.80 |
|  | **E4** | 87.46 | 6.30 | 73.50 | 112.00 | 86.00 | 38.50 | 7.10 |
|  | **E5** | 66.72 | 6.49 | 52.50 | 92.50 | 64.00 | 40.00 | 10.90 |
| DTM (days) | **E1** | 135.27 | 1.64 | 132.00 | 143.00 | 134.00 | 11.00 | 1.21 |
|  | **E2** | 134.85 | 2.87 | 108.00 | 153.00 | 133.00 | 45.00 | 2.10 |
|  | **E3** | 134.84 | 1.94 | 126.00 | 145.00 | 134.00 | 19.00 | 2.70 |
|  | **E4** | 133.63 | 8.07 | 124.00 | 156.00 | 133.00 | 32.00 | 6.00 |
|  | **E5** | 105.28 | 4.64 | 95.00 | 124.00 | 105.50 | 29.00 | 4.71 |
| PH (cm) | **E1** | 101.98 | 12.00 | 71.25 | 139.50 | 94.25 | 68.25 | 11.77 |
|  | **E2** | 109.27 | 14.01 | 69.00 | 147.00 | 100.00 | 78.00 | 12.81 |
|  | **E3** | 105.62 | 11.59 | 70.13 | 135.50 | 98.50 | 65.38 | 10.97 |
|  | **E4** | 113.18 | 18.17 | 70.00 | 162.50 | 102.00 | 92.50 | 16.00 |
|  | **E5** | 94.45 | 15.00 | 56.00 | 129.00 | 82.50 | 73.50 | 11.80 |
| SL (cm) | **E1** | 10.40 | 1.59 | 5.60 | 15.33 | 10.67 | 9.67 | 15.26 |
|  | **E2** | 10.33 | 1.40 | 5.60 | 14.33 | 10.17 | 8.67 | 13.55 |
|  | **E3** | 10.33 | 1.51 | 5.60 | 16.17 | 9.67 | 10.50 | 14.60 |
|  | **E4** | 11.97 | 1.51 | 7.80 | 15.85 | 12.20 | 8.05 | 12.57 |
|  | **E5** | 13.30 | 2.23 | 7.00 | 20.00 | 13.00 | 13.00 | 16.74 |
| Awn_L (cm) | **E1** | 7.46 | 1.83 | 0.00 | 13.00 | 13.00 | 13.00 | 25.40 |
|  | **E4** | 7.03 | 2.15 | 0.00 | 19.50 | 19.00 | 19.50 | 29.30 |
| Leaf_L (cm) | **E1** | 28.48 | 3.48 | 20.00 | 41.00 | 25.00 | 21.00 | 12.20 |
|  | **E4** | 27.48 | 3.95 | 16.25 | 41.40 | 29.00 | 25.00 | 14.30 |

E1- average of two replications at ICAR-IIWBR, Karnal (2016); E2 - average of two replications at Seed Farm, Karnal (2016); E3- average of two locations of Karnal (2016); E4 - average of two replications at ICAR-IIWBR, Karnal (2017) and E5 - average of two replications at Bhavnagar, Gujarat (2016); Std.Dev: Standard deviation; Env: Environment; CV: coefficient of variance.
