# Supplementary material for: Uncovering Genomic Regions Associated With 36 Agro-Morphological Traits in Indian Spring Wheat Using GWAS
Source: Front Plant Sci. 2019 Apr 25;10:527. doi: 10.3389/fpls.2019.00527 (PMC6511880; doi:10.3389/fpls.2019.00527)
Supplement: Supplementary file 10 [file Table_4.docx]

**Table S4: Analysis of variance (ANOVA) of the four traits (DTH, DTM, SL, PH) in 260 wheat genotypes**

|  |  | ***df*** | **DTH** | **DTM** | **SL** | **PH** |
| --- | --- | --- | --- | --- | --- | --- |
| **Type III SS** | **Genotype** | 259 | 61054.9674 | 21644.7056 | 2893.3087 | 400470.4864 |
|  | **Env^a^** | 3 | 222087.1935 | 352703.9700 | 2072.4494 | 87164.7654 |
|  | **Rep** | 1 | 461.1456 | 26.8965 | 8.9236 | 3381.6064 |
|  | **Genotype*Env** | 777 | 14847.2061 | 20082.4981 | 1848.7188 | 65355.3357 |
|  | **Genotype*Env*Rep** | 780 | 8589.6913 | 4567.3681 | 876.5471 | 30488.2173 |
| **Mean Square** | **Genotype** | 259 | 235.7335 | 83.5703 | 11.1711 | 1546.2181 |
|  | **Env^a^** | 3 | 74029.0645 | 117567.9900 | 690.8165 | 29054.9218 |
|  | **Rep** | 1 | 461.1456 | 26.8965 | 8.9236 | 3381.6064 |
|  | **Genotype*Env** | 777 | 19.1084 | 25.8462 | 2.3824 | 84.1124 |
|  | **Genotype*Env*Rep** | 780 | 11.0124 | 5.8556 | 1.1281 | 39.0875 |
| **F Value** | **Genotype** | 259 | 13.5** | 2.87** | 5.15** | 16.95** |
|  | **Env^a^** | 3 | 3874.17** | 4548.75** | 290.01** | 345.43** |
|  | **Rep** | 1 | 47.29** | 2.66 | 9.16* | 69.96** |
|  | **Genotype*Env** | 777 | 1.74** | 4.41** | 2.11** | 2.15** |
|  | **Genotype*Env*Rep** | 780 | 0.53 | 0.24 | 0.72 | 0.38 |
|  | **Broad Sense heritability (H**^2^) |  | 0.83 | 0.41 | 0.61 | 0.85 |

**p<*0.05*, **p<*0.0001*,* Env = Environment, Rep = replication, SS = sum of squares, *df* = degree of freedom, DTH =days to heading, DTM = days to maturity, SL = Spike length, PH = plant height.
